# Sustainable One‐Pot Electrochemical Approach for Entrapment of Multi‐Enzymes and Biocompatible Redox Mediator

**DOI:** 10.1002/cplu.202400577

**Published:** 2025-02-20

**Authors:** Hathaichanok Seelajaroen, Felix Mayr, Dominik Wielend, Munise Cobet, Christoph Ulbricht, Niyazi Serdar Sariciftci, Serpil Tekoglu

**Affiliations:** ^1^ Linz Institute for Organic Solar Cells (LIOS) Institute of Physical Chemistry Johannes Kepler University Linz Altenberger Str 69 A-4040 Linz Austria

**Keywords:** Bioelectrocatalysis, CO_2_ reduction, Dehydrogenases, Enzyme immobilization, Neutral red, Polyypyrrole

## Abstract

Enzyme immobilization is regarded as a key factor for their effective utilization in various fields such as biofuel production, wastewater treatment, and biosensors. Designing new electrodes with biocompatible support matrices is essential to improve the stability in bioelectrocatalysis. Modified carbon felt electrodes were prepared and tested as bioelectrocatalyst under mild conditions – aqueous media at room temperature and basically neutral pH. Co‐immobilization of dehydrogenase enzymes and neutral red (NR) dye at carbon felt electrodes was successfully achieved during electropolymerization of pyrrole in a facile one‐step approach. Neutral red was incorporated to operate as a redox mediator supporting efficient electron transfer to the enzymes’ active sites. Electrodes modified with alcohol dehydrogenase (ADH) have been employed to reduce acetaldehyde to ethanol in a chronoamperometic setting with Faradaic efficiency (FE) of up to **33 %**. By incorporating the cascade reaction with three enzymes – ADH, formate dehydrogenase, and formaldehyde dehydrogenase – an electroreduction sequence could be established to produce methanol from CO_2_ reaching a FE of **10 %**. The proposed approach shows good stability. Together with the simple implementation and application, this process is promising for employment in enzymatic electrocatalysis.

## Graphical abstract

Novel enzyme‐modified carbon felt electrodes are constructed for efficient bio‐electrocatalysis. Simultaneous electrodeposition of pyrrole and neutral red provides a facile one‐step approach for enzyme immobilization. The biocompatible conductive polymer matrix performs for the entrapment of dehydrogenase enzymes on the electrode and ensures sufficient electron transfer during direct electron transfer without cofactor.

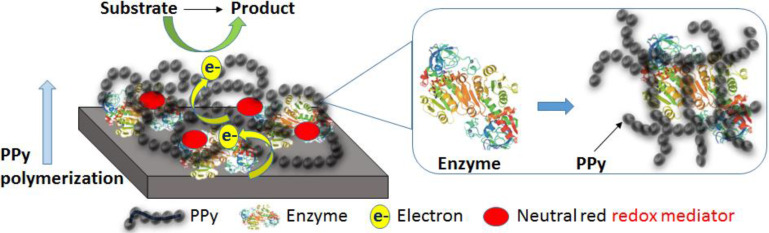

## 1 Introduction

Biomolecule immobilization has drawn tremendous attention in different fields for applications such as wastewater treatment, immunosensors, biofuels and carbon dioxide (CO_2_) utilization, inter alia.^[1]^ In particular, CO_2_ conversion through electrochemical reactions is favorable for the atmospheric CO_2_ mitigation in order to retard the adverse effect on global warming.^[2]^ CO_2_ conversion to one‐carbon (C1) compounds such as methanol, methane, CO and formate could provide the basis for valuable renewable fuels.^[3]^

The enzymatic electrochemical reduction of CO_2_ promises a controllable process with its high recognition due to high specificity and a simple work flow under mild and safe operational conditions.[^4^, ^5^] On the other hand, the enzyme recovery, purification, and regeneration are the limiting factors in homogenous biocatalysis.^[5]^ Efficient electron transfer reaction between enzyme and electrode is herewith an important phenomenon for the realization of redox enzymes to develop enzymatic electrocatalysis for fuel cells and biosensor.^[6]^ Therefore, the third generation electrode platform with direct electron transfer (DET) is considered superior to the second generation electrodes with mediated electron transfer (MET). In DET, direct electronic coupling between an electrode and enzymes is created by a suitable entrapment technique which plays a crucial role on the biomolecules’ stability during redox processes. The adherence of enzymes on electrode surface allows operational potentials closer to formal potential (E°) of the redox enzyme while reducing possible interfering reactions.^[6]^ In addition, there is no diffusion of mediator in solution for an immobilized mediator‐based system compared to the second generation platform that relies on dissolved mediators in electrolyte solution.^[7]^

Non‐conductive polymer matrix may affect DET process by hindering biological electron transfer mechanism between the active site of enzyme and electrode.^[8]^ In this context, conductive polymers such as polyaniline, polypyrrole and polythiophene have been intensively investigated for enzyme immobilization to increase the catalytic activity of enzymes in biosensors and for biofuel production.[^9^, ^10^] Polypyrrole (PPy) has drawn particular attention owing to relatively stable electrical conductivity as well as facile electrochemical deposition under biocompatible conditions in aqueous environment at low oxidation potentials and neutral pH.[^11^, ^12^, ^13^] Besides its good conductivity, electrochemically synthesized PPy films exhibit an attractive feature with its high adherence to common electrode surfaces.^[13]^

Another critical challenge is mild operational conditions of biocatalysts that can restrict CO_2_ conversion to C1 products. An efficient electron transfer during electrolysis can increase the rate of bio‐electrocatalysis. Stabilizing the enzyme on the electrode surface can provide adequate electron supply in addition to solving the byproduct and purification problem. Yet, it remains challenging to entrap the enzyme on the electrode for rapid electron injection while maintaining its activity and a reasonable stability. Mediators have been involved as redox shuttles in the mechanism to further increase the electron transfer.[^4^, ^14^]

**Figure 1:**
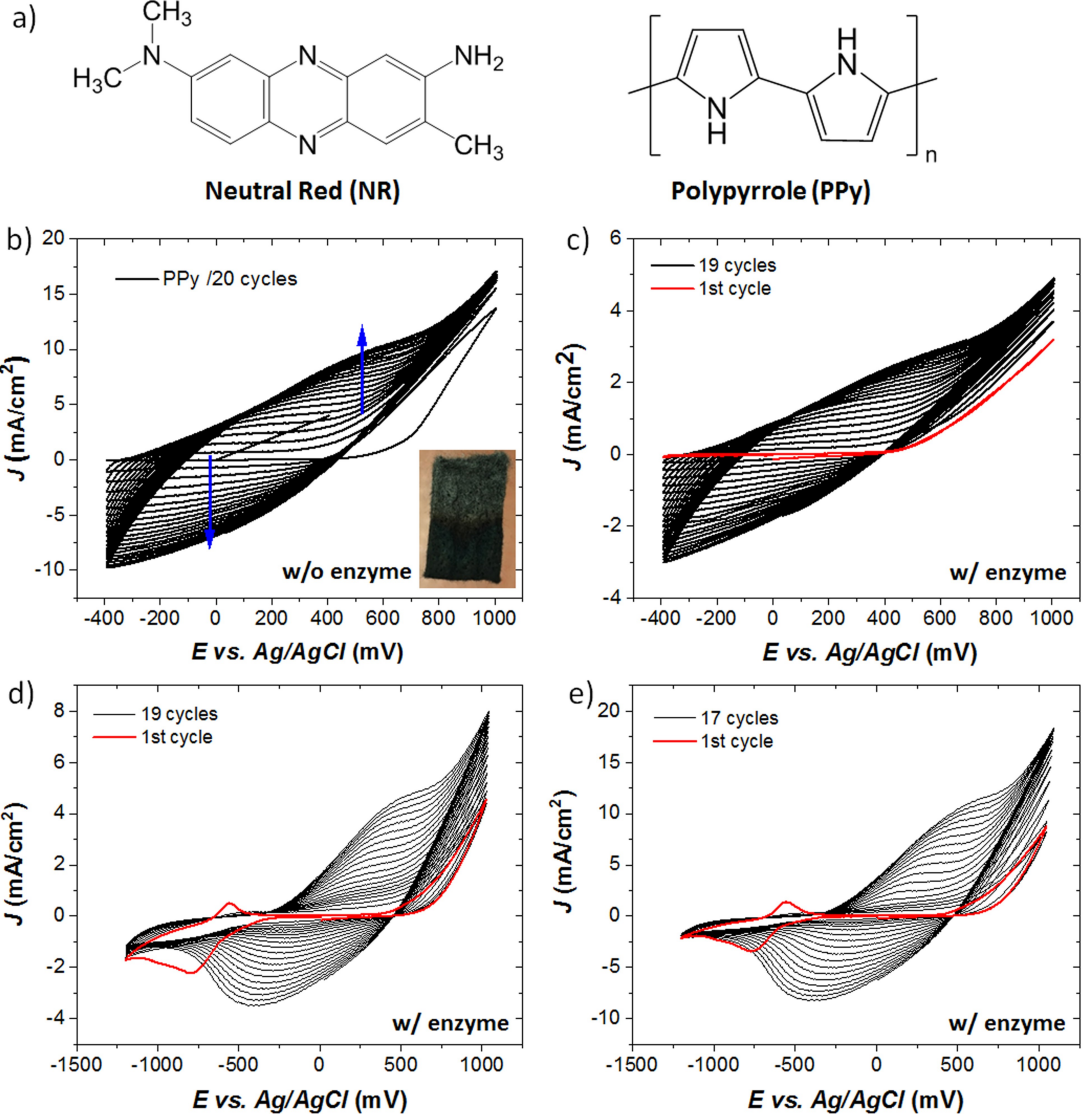
a) Molecular structures of Neutral Red (NR) and polypyyrole (PPy). Potentiodynamic polymerization of 0.3 M pyrrole in 0.1 M PBS solution after 20 cycles at a scan rate of 50 mV/s, **b)** without enzyme and **c)** with ADH enzyme adhered on the carbon felt electrodes. Electropolymerization of 0.15 M pyrrole with the addition of **d)** 0.2 mM neutral red and **e)** 0.4 mM neutral red into the electrolyte solution prior to polymerization. Inset image: modified CF electrode showing PPy covered the bottom of the electrode (dark color).

Redox mediator molecules, such as methyl viologen, benzyl viologen, neutral red (NR), and anthraquinone 2,6‐disulfonate have been involved in transferring electrons to enzymes via direct or indirect electron transfer.[^15^, ^16^] Among the redox shuttles, the phenazine dye neutral red (3‐amino‐7‐dimethylamino‐2‐methylphenazine hydrochloride) shown in Figure 1a stands out with its distinguished properties such as low toxicity, low reduction potential, and working at the physiologically relevant range pH 6.8–8.^[17]^ The low reduction potential peak at −525 mV_Ag/AgCl_ is close to the standard potential of the biological redox cofactor nicotinamide adenine dinucleotide hydride (NADH; −515 mV_Ag/AgCl_).^[17]^ Therefore, NR as well as its polymeric form are frequently deployed as redox mediator for microbial bio‐electrocatalysis.[^17^, ^18^, ^19^] Apart from the studies with microbial fuel cells, NR has been utilized in the enzymatic bio‐electrocatalysis.[^20^, ^21^] NR performs as biocompatible redox mediator without inhibitory effect on the enzymatic performance.^[22]^ Moreover, NR‐modified non‐conjugated polymer has been reported for redox‐polymer‐based enzymatic electrodes for nitrogen fixation.^[21]^ NR has been immobilized within conductive polyaniline (PANI) on carbon paper to provide a platform for microbial electrosynthesis.^[23]^ Simultaneous co‐immobilization of enzymes with redox mediators in PPy matrix has been previously realized on ITO‐glass and platinum electrodes for amperometric biosensors and biofuel cells, respectively.[^24^, ^25^] However, so far there is no report of immobilized NR within a conductive polymer support on carbon felt used in CO_2_ reduction.

In this study, we highlight the modified electrodes composed of a polypyrrole support matrix, a redox mediator and dehydrogenase enzymes as prepared all in one‐step. The proposed approach can provide an alternative hybrid electron transfer mechanism as enzyme and redox mediator are entrapped together within a polymeric conductive support matrix on top of porous carbon electrode (e. g. carbon felt). Neutral red was investigated as immobilized redox mediator within polypyrrole films, in order to increase electron injection to the enzymes’ active site without hampering biocompatibility and conductivity. Simultaneous electrodeposition of PPy and NR as well as the immobilization of dehydrogenase enzymes were successfully achieved via cyclic voltammetry at mild conditions in aqueous environment (pH 7.4) at room temperature. The morphology of the modified electrodes was investigated by scanning electron microscopy (SEM). X‐ray photoelectron spectroscopy (XPS) and Raman spectroscopy were conducted to analyze the chemical structure of the electrodeposited films. The enzyme‐modified electrodes were tested for acetaldehyde and CO_2_ conversion. Liquid‐injection gas chromatography was used to detect formed products. UV‐Vis spectroscopy was used to check for potentially leached neutral red within the aqueous electrolyte after the electrolysis. The Faradaic efficiency was determined to 33 % and 10 % for ethanol and methanol production, respectively. In both cases, the immobilized enzymes were found to retain functionality and exhibited high stability as biocatalyst without the deployment of NADH cofactor.

## 2 Results and Discussion

### 2.1 Cyclic Voltammetry for Synthesis of Modified Electrodes

Electrochemical polymerization was employed as simple technique with controllable process by changing the preparation conditions. The synthesis method involved in our previous publication for enzyme‐free electrocatalysis was adapted for carbon felt electrode.^[26]^ Carbon felt (CF) was selected as working electrode due to its porous 3D surface. The porous carbon‐fiber electrodes provide a large surface area for enzyme entrapment and immense pore volume for CO_2_ molecules which has a significant influence on the conversion efficiency of CO_2_ reduction.[^27^, ^28^] The electrochemical synthesis was performed in 0.1 M phosphate buffered saline (pH 7.4) containing NaCl, KCl, Na_2_HPO_4_ and KH_2_PO_4_ with the appropriate concentrations detailed in the experimental section. The molecular structures of neutral red (NR) and polypyyrole (PPy) were depicted in Figure 1.

PPy was electrochemically synthesized on CF substrates by sweeping the potential between −0.4 V and +1.0 V. Cycle numbers and monomer concentrations were varied to optimize the process.

The results of the optimization are shown in the supplementary information (Figure S1a–c). There are different experimental formulations for the preparation of PPy, however, oxidative electrochemical polymerization is usually initiated at potentials above +0.6 V versus Ag/AgCl.^[29]^ The electropolymerization relies on the condensation of pyrrole monomer units. In parallel, negatively charged counter ions must be present in the solution as counterion to maintain doping through the positively charged PPy backbone. The polymer in as synthesized form is obtained in the corporation of anions from PBS electrolyte. PBS is used to preserve a physiological pH in which the dehydrogenase enzymes can function without interfering the process (inhibition of enzymes or toxicity).^[30]^

The oxidative electropolymerization of pyrrole was optimized by monomer concentrations of around 0.15 M and 0.3 M. The emulsion was stirred vigorously prior to synthesis. Enzyme modified electrodes were constructed with the optimized monomer ratio and the number of cycles in cyclic voltammetry. In‐situ electropolymerization of 0.3 M pyrrole was successfully achieved with and without alcohol dehydrogenase (ADH) adhered on CF electrode (Figure 1c–d). The synthesis was completed after continued cycling between the neutral and p‐doped form in the range of −0.4 V to +1.0 V vs Ag/AgCl with the sweep rate of 50 mV/s for 20 cycles at room temperature with an average of 21 °C. The red curves correspond to the first cycle of polymerization. In both cases, accumulation of the conductive polymer film on the electrode surface results in an inclined current level while the polymerization proceeds. It leads to the deposition of thin‐films from the monomer solution onto CF with the surface area of ~1 cm^2^ as seen in the inset image of Figure 1c. For the enzyme modified electrode in Figure 1d, the CF electrode was immersed into 6 mg mL^−1^ enzyme solution overnight prior to the synthesis. The current density dropped with the adherence of enzymes onto the electrode. It can be attributed to the pyrrole monomers may not be able to fully diffuse on CF during polymerization in presence of enzymes. All CV results were in good agreement with the literature.[^26^, ^29^]

We extended our work for the polymerization with the optimized concentration of pyrrole (0.15 M) for co‐immobilization of redox mediator and ADH on carbon felt electrode (Figure 1e–f). Modification of carbon felt with ADH was achieved by a tailored method which entails soaking of CF in electrolyte solution which pyrrole and redox mediator dissolved, and followed by rapid simultaneous polymerization. The film was obtained upon repetitive potential sweeps covering the reductive potential of neutral red and oxidative potentials of PPy in the range of −1.2 V to +1.0 V vs Ag/AgCl with the sweep rate 50 mV/s with various cycles depending on the monomer concentration of neutral red. The number of sweeping cycles were adjusted as 20 and 18 for the concentration of neutral red 0.2 mM and 0.4 mM, respectively. Both electrodes exhibit larger current response comparing to the CF/ADH/PPy electrode without NR. Higher NR amount in the PPy results in a higher current density. The reduction and oxidation peaks of NR are prominent on the first cycle of electropolymerization. The presence of distinguished peaks for NR‐modified PPy electrodes in the first cycles indicate the difference between the control electrodes with pristine PPy. When the cycle number increases, PPy reduction peak becomes pronounced and showed larger current response. The enhanced current densities of the modified electrodes represent improved electrochemical activities. The reduction potential of NR is −521 mV vs Ag/AgCl, which is close to the standard redox potential of NR.^[17]^

### 2.2 XPS and RAMAN Spectroscopic Characterization

**Figure 2:**
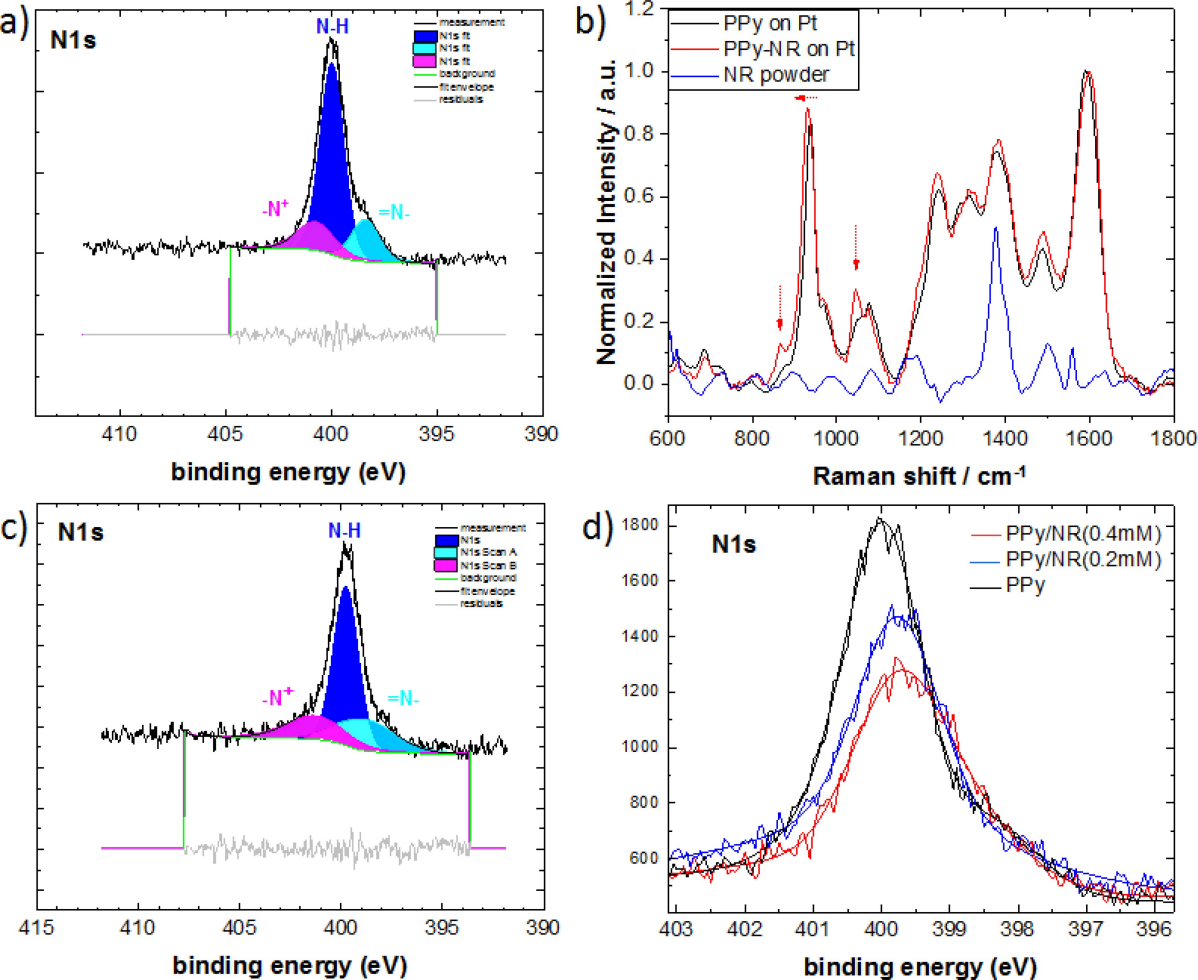
XPS spectrum of polypyrrole with and without adding neutral red (NR). Peak deconvolution for the N1s for **a**) PPy and **c**) PPy‐NR (0.4 mM). **b**) Raman spectrum of PPy (black), PPy‐NR (red) and neutral red powder (blue) measured at an excitation wavelength of 1064 nm. **d**) Comparison of XPS peak deconvolution for the N1s of PPy, PPy‐NR(0.2 mM), and PPy‐NR(0.4 mM).

X‐ray photoelectron spectroscopy (XPS) and Raman spectroscopy were performed to examine the chemical structures of the materials. XPS confirmed the presence of the C, O, and N elements in the samples. The deconvolution of the N1s region is depicted in Figure 2. The components at ~400 eV are assigned to the neutral amine nitrogen (N−H) and the higher binding energy at 401 eV are related to the positively charged nitrogen (−N^+^). The component with the lowest binding energies at the range of 398.5 eV corresponds to the imine nitrogen (=N−).[^31^, ^32^] The presence of –N^+^, indicates that the PPy component is doped.^[33]^

In comparison to the PPy reference sample, the N1s core level XPS spectrum of the PPy sample with neutral red shows a markedly increased contribution of a lower binding energy peak component centered at ~*398* eV in comparison to the main component at 399.9 eV (Figure 2d). This may be attributed to the more electron‐rich nitrogen atoms in the phenazine moiety in neutral red. Another contribution may be a difference in the doping level of PPy induced by the addition of NR, resulting in a larger fraction of neutral pyrrolic nitrogens observed at lower N1s binding energy. A similar shift has been observed in the neutral nitrogen peak for PPy‐chloranil complex with the addition of organic acceptor in PPy via simultaneous chemical polymerization.^[34]^

The deconvolution of the C1s region at the range of 284 to 290 eV is depicted in Figure S2. A strong dominant peak at 284.7 eV is assigned to sp^2^ hybridized carbon present in aromatic ring of PPy.[^35^, ^36^] This peak also belongs to carbon atoms present in the aromatic ring of neutral red. Other carbon peaks at binding energy of 286.4 eV and 287.8 eV indicate the possible formation of C−OH/C=N/=C−NH^+^.^[36]^ The peak at 290.3 eV can be attributed to the π–π^✶^ satellite, which is commonly found in aromatic systems.^[37]^ The survey for a complete elemental analysis with C, O, and N ratios can be found in the supplementary information.

Raman spectroscopy was recorded to compare the chemical structures of PPy, PPy‐NR and pristine NR and the results are shown in Figure 2b. Raman analysis of PPy and PPy‐NR with various bands vibrations between 680 cm^−1^ and 1690 cm^−1^ is consistent with previously reported values for PPy. The predominant band vibrations at 938 cm^−1^, 1075 cm^−1^, 1241 cm^−1^, 1376 cm^−1^, and 1590 cm^−1^ correspond to the C−C ring deformation (bipolaron), C−H in‐plane deformation (oxidized PPy), antisymmetric C−H in‐plane bending, antisymmetric C−N stretching, and C=C interring stretching for PPy, respectively.[^31^, ^38^] The band vibration at 938 cm^−1^ shifts to 930 cm^−1^ for PPy‐NR. Additionally, the peak shoulders at 884 cm^−1^ and 1048 cm^−1^ observed for pristine PPy appear as more pronounced and slightly shifted to 865 cm^−1^ and 1044 cm^−1^ for PPy‐NR. In accordance to previous reports, these changes in the characteristic peaks can be attributed to changes of the doping level in PPy, in conformity with the observations from XPS.[^38^, ^39^] A similar shift in Raman spectra has been observed after a different phenazine dye safranin‐O incorporated in PPy.^[40]^

### 2.3 Scanning Electron Microscopy Analysis

**Figure 3:**
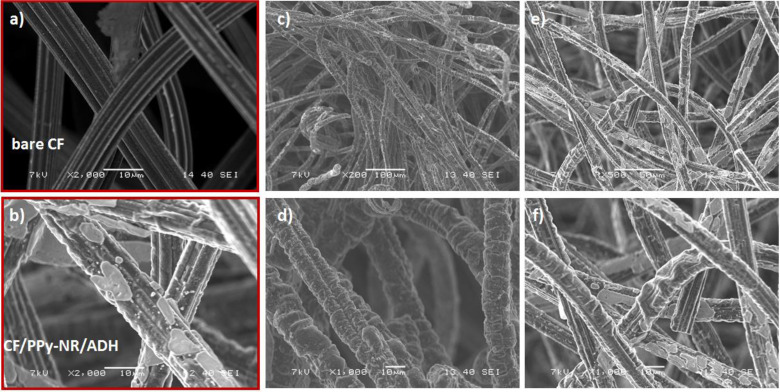
SEM images of pristine and modified carbon felt electrodes with ADH enzyme. **a**) Bare and **b**) modified carbon felt with 2,000× magnification. **c**–**d**) CF/PPy/ADH with 200× and 1,000× magnifications, respectively. 0.3 M pyrrole was polymerized on CF over 20 cycles. **e**–**f**) CF/PPy‐NR/ADH with 200× and 1,000× magnifications. Electrode was modified with the polymerization of 0.15 M pyrrole over 18 cycles in the presence of 0.4 mM neutral red.

Scanning electron microscope (SEM) images were used to examine the surface of the electrodes. Topography measurements on carbon felt electrodes reveals the morphological changes for different modifications (Figure 3). The bare carbon felt electrode possessed a clean surface with fibrous structure (Figure 3a). The CF fibers were covered with PPy nanofilm after electropolymerization. The SEM images of the carbon felt electrode with immobilized ADH enzyme are shown in Figure 3b–f for surface analysis. The unmodified carbon felt electrode has a smooth surface with a fiber diameter that varied between 8 μm and 10 μm (Figure 3a). A relatively rough surface is observed upon polymerization. CF fibers were covered with the polymer resulting in an average diameter increased to 12–14 μm. The PPy films are composed of a wrinkled globular structure. The high magnification SEM micrographs reveal that the PPy layer is covered vertically on the CF fibers forming a surface with the three‐dimensional conductive carbon skeleton with PPy including the enzymes. The electrode surface can be increased with the porous PPy film covered fibers in contrast to the flat surface.[^26^, ^28^]

SEM images of the modified electrodes after washing in PBS solution for 1 h and 24 h are depicted in (Figure S3a–d). After 16 h electrochemical reduction, SEM was performed to detect changes in the surface morphology. No cracks or exfoliation were observed suggesting robust attachment of PPy on CF (Figure S3e–f). Enzymes were not detected by SEM analyses due to their small size in nanometer level, whereas CF fibers were in the range of micrometers. According to Eriksen et al.,^[41]^ starting from the weight of 74 kDa for FDH and 144 kDa for ADH, the diameter of dehydrogenase enzymes is smaller than 10 nm.^[42]^

### 2.4 Conversion of Acetaldehyde to Ethanol by ADH

The ADH‐modified electrodes were tested for acetaldehyde reduction using the sacrificial cofactor NADH as an electron and proton source without applying any voltage. The catalytic reaction was initiated by NADH addition (1×10^–6^ mol) and after 16 h, liquid samples were collected and analyzed with gas chromotography for ethanol production (Table S1). A maximum conversion efficiency of ~41 % was calculated for the chemical conversion of acetaldehyde to ethanol using modified electrode CF/PPy‐NR/ADH. To assess any potential catalytic contribution from pristine poly(neutral red) (PNR) in immobilizing ADH units on carbon felt, a control sample was prepared by electropolymerizing 1 mM NR to encapsulate the enzyme on CF. A CE of 5.8 % was obtained for the designated sample of CF/PNR/ADH, indicating that the pristine PNR was insufficient to retain the enzyme on the electrode surface. These findings further confirmed that the catalytic activity of CF/PPy‐NR/ADH stemmed from the hybrid electron transfer mechanism facilitated by enzyme immobilization. An additional control CF/PPy‐NR without ADH, exhibited no product formation.

**Figure 4:**
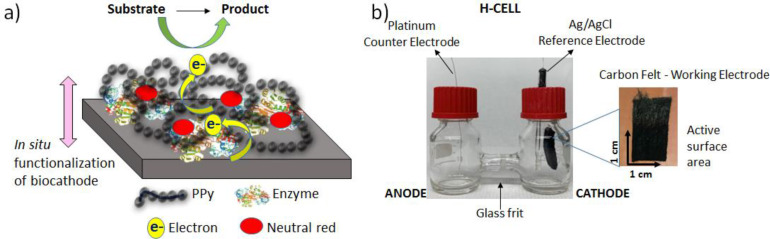
**a**) A schematic illustration of electron transfer accompanying the biocatalytic process with enzyme‐modified electrode. **b**) Two‐compartment electrochemical setup with a Pt counter electrode, an Ag/AgCl (3 M KCl) reference electrode and an enzyme modified working electrode (surface area=1 cm^2^) for electrocatalysis.

Electrochemical methods such as cyclic voltammetry (CV), chronoamperometry have been widely utilized to detect the catalytic activity of enzyme‐modified electrodes. The enzyme is entrapped on the carbon felt electrode within a thin‐film of polymer that facilitates efficient electron transfer between the enzyme and the electrode during the biocatalytic process (Figure 4a). In this schematic, the enzyme physically entrapped the polymer matrix provides conducting support to the enzyme via electron hopping mechanism through redox sites and accessing to the active side of enzyme. The redox‐mediator sites presented (red dots on the sketch) within polypyrrole matrix to further increase the electron transfer. Then, the biocatalyst enables the conversion of substrate to product. All the electrolysis procedures were completed in a two‐compartment electrochemical cell (H‐cell) equipped with three‐electrode system (Figure 4b). Prior to the electrolysis, the electrochemical performance of the enzyme‐modified electrodes is studied by conducting CV analysis under N_2_. Cyclic voltammograms were recorded in 0.1 M PBS (pH 7.4) for the ADH‐modified electrode as working electrode in the cathode side of H‐cell by sweeping the potentials between 0 V and −1.2 V.

**Figure 5:**
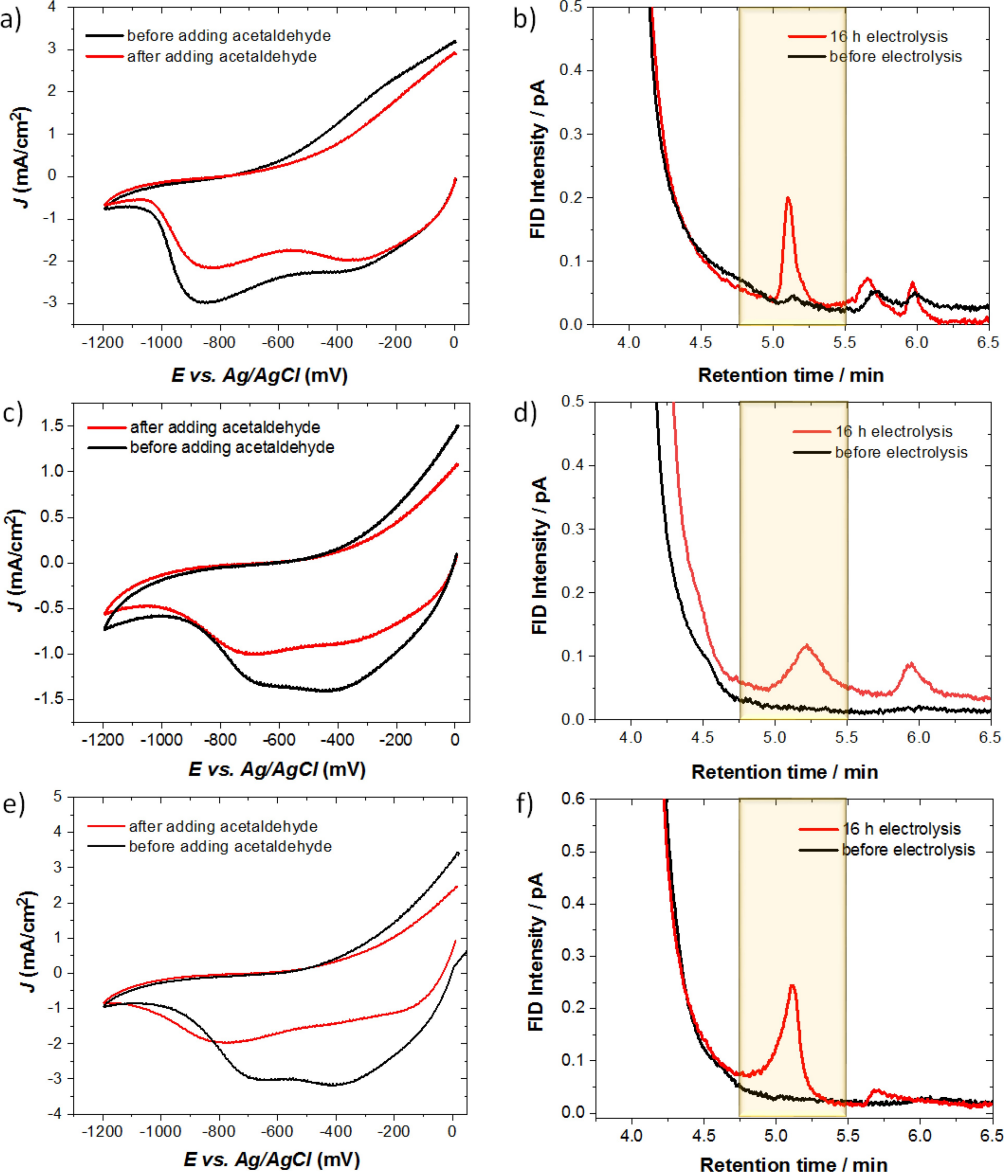
Cyclic voltammograms of modified electrodes in 0.1 M PBS (pH 7.4) before and after adding acetaldehyde at an applied voltage between 0 V and −1.2 V with the scan rate of 25 mV/s: **a**) CF/PPy/ADH, **c**) CF/PPy‐NR(0.2 mM)/ADH, **e**) CF/PPy‐NR(0.4 mM)/ADH. Chromatograms for the ethanol production analysis before and after 16 h electrolysis: **b**) CF/PPy/ADH, **d**) CF/PPy‐NR(0.2 mM)/ADH, **f**) CF/PPy‐NR(0.4 mM)/ADH. (FID: Flame ionization detector intensity).

Electrochemical characterization of ADH‐modified electrodes with and without NR before and after adding acetaldehyde into the system is shown in Figure 5. Two couple of redox waves on the voltammogram are attributed to the reduction peaks of polypyrrole. All the modified electrodes were stable in the cathodic region up to −1.2 V vs Ag/AgCl for the reduction of acetaldehyde. CF/PPy‐NR(0.4 mM)/ADH electrodes show an irreversible reduction peak at around −0.4 V with nearly −1.5 mA/cm^2^ current density from the polymer redox behavior. By increasing the immobilized NR amount, the identical reduction peak is observed with higher current density over −3.0 mA/cm^2^. The associated peak current indicates that maximum reactive species at the electrode interface, whereas the onset potentials remain same.

Considering that PPy is synthesized oxidatively, the conductive form of it can be reduced to the neutral form at negative potentials. John and Wallace studied the reduction and oxidation of polypyrrole and observed the reduction of oxidized PPy to the neutral form at −800 mV.^[43]^ The neutral form with a significant lower electrical conductivity can hinder the reduction process of CO_2_ at negative overpotentials. In this context, immobilized n‐type semiconductor neutral red as a secondary electrocatalytic material within the PPy matrix can be beneficial to improve electron transfer between the electrode and the enzyme. The improved catalytic performance was reported elsewhere, where two electrocatalytic materials are combined to the enhanced electron transfer rate provided by the interactions between two electrocatalytic components.^[44]^ Similarly, neutral red and methyl viologen modified polyaniline electrodes indicate the redox mediator‐modified electrodes improved the electrochemical activities.^[23]^

Chronoamperometric electrolysis were performed for 16 h under applied constant potential, −1.0 V, for the reduction of acetaldehyde (Figure 5). The Faradaic efficiency (FE) is calculated using following formula in the experimental section. After the electrolysis, 33 % ethanol production was obtained for the immobilized system with high amount of NR (Figure 5f), whilst the ethanol yield of 14 % was detected from catalysis (Figure 5d) with low amount of NR for an equivalent system with enzyme modified electrode (using the same concentration of enzyme). The ethanol production is significantly improved comparing to the reaction with pristine PPy matrix with ADH showing FE 9 % (Figure 5b). The highest production of 11.5 ppm was obtained for the CF/PPy‐NR(0.4 mM)/ADH. PPy with closely packed polymer chains as support matrix retains the enzyme on the electrode. While PPy worked as support matrix and sufficient electrical conductivity at the interface of the CF electrode and enzyme, NR performed as redox mediator to improve an efficient charge injection into the active sites. Current‐time curves of chronoamperometric electrolysis for all the electrodes at the applied constant potential of −1.0 V for 16 h are depicted in the supplementary information (Figure S4a–c).

UV‐Vis spectra were recorded (SI, Figure S5) for the washing solution of modified electrode (CF/PPy‐NR/ADH) and the sample solution after completed electrolysis to examine any residue of the neutral red. The absorption spectrum shows maxima at the wavelengths of 530 with a shoulder at 456 nm of neutral red in PBS solution during washing steps. The data is consistent with the previously published results exhibiting both absorption peaks at 455 nm and 525 nm for NR and its cationic form NR^+^H, respectively.^[45]^ The spectra for the sample solution after the electrolysis exhibits no residual effect of neutral red during the electrolysis to confirm heterogeneous catalysis. No noticeable peaks detected in absorption spectra for the electrolyte tested after the electrolysis, suggesting that the modified CF electrode was stable.

As control sample, the catalytic activity of pristine CF/PPy without enzyme was tested as catalyst and the results are shown in suppl. data, Figure S7. No catalytic activity was detected for pristine PPy film on CF. Further experiment was organized with pristine poly‐neutral red (PNR) as support matrix. NR was polymerized on CF electrode to retain ADH enzyme (Figure S8). However, PNR polymerization resulted in much lower coverage on CF fibers compering to PPy to entrap the enzyme on the electrode efficiently (Figure S9). The system resulted in low amount of immobilized enzyme and consequently no product formation was detected after electrolysis.

### 2.5 Modified Electrodes with Multi‐Enzymes (Dehydrogenases)

As the second objective of this work, the optimized polymerization method was applied for the stabilization of multi‐enzyme system. Multi‐enzyme electrocatalysis is a very attractive approach and commonly used for CO_2_ conversion to unique products due to high specificity of enzymes.[^8^, ^42^, ^46^]

**Figure 6:**
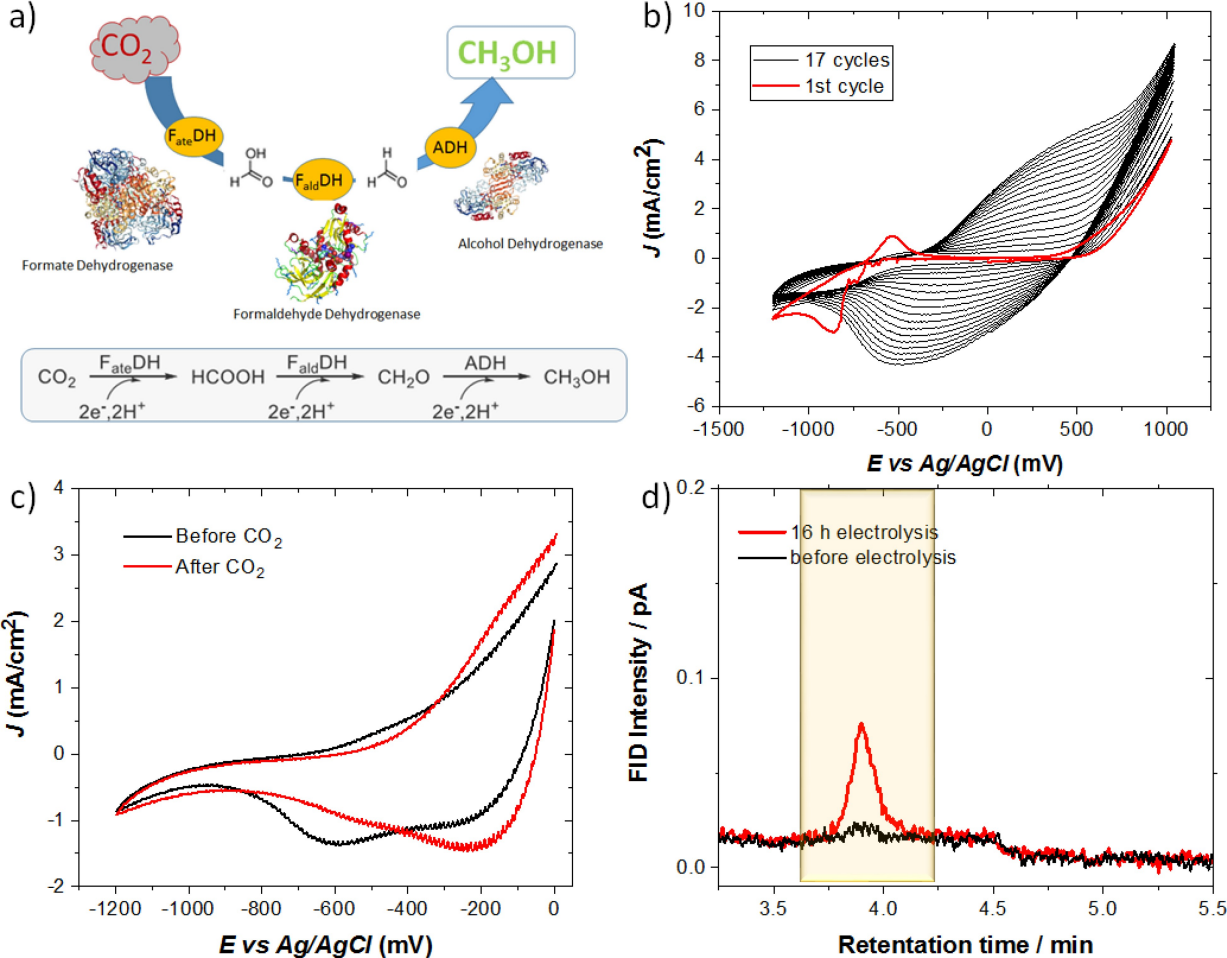
**a**) A schematic illustration of multi‐enzymatic cascade reaction steps for CO_2_ conversion. **b**) CV of preparation of the CF/PPy‐NR/DHs electrode. 0.15 M pyrrole and 0.4 mM NR were electrochemically deposited by potential sweep between −1.2 V and +1.0 V after 18 cycles at the scan of 50 mV/s in 0.1 M PBS (pH 7.4). **c**) Cyclic voltammograms of CF/PPy‐NR/DHs in 0.1 M PBS (pH 7.4) before and after purging system with CO_2_ at an applied voltage between 0 V and −1.2 V with the scan rate of 25 mV/s. **d**) Chromatograms for the methanol production analysis before and after 16 h electrolysis.

In‐situ production of methanol from reduced CO_2_ is achieved through the cascade reaction composed of three oxidoreductase enzymes namely formate dehydrogenase (FDH), formaldehyde dehydrogenase (F_al_DH), and alcohol dehydrogenase (ADH). Figure 6a depicts the schematic illustration of the reaction route with the intermediate products and the number of electrons required for each step of the reduction process. For the conversion of CO_2_ to methanol, 6 electrons are involved in total for the cascade reaction. All dehydrogenase enzymes and NR were entrapped in electropolymerized PPy which was optimized to stabilize ADH in the previous section. Prior to synthesis of PPy‐NR, CF electrode was soaked into the enzyme solution with the concentration of 7 mg mL^−1^ for 20 h overnight.

0.15 M pyrrole was electrochemically polymerized with addition of 0.4 mM NR were by sweeping the potential between −1.2 V to +1.0 V vs Ag/AgCl at the sweep rate 50 mV/s within 18 cycles. The reduction and oxidation peaks of NR are prominent on the first cycle of electropolymerization in Figure 6b. The surface topography of the modified electrode was investigated by SEM. The electron microscopy dataset indicates the difference of the CF fibers in nanometer scale. The CF fibers were covered with the thin layer of polymer uniformly confirming the porous 3D structure network (Figure S6).

### 2.6 Conversion of CO_2_ to Methanol by Multi‐Enzymes

The electrochemical characteristics of CF/PPy‐NR/DHs electrodes were analyzed for CO_2_ reduction to methanol in aqueous media with 0.1 M PBS (pH 7.4) prior to the electrolysis. The voltammograms are depicted in Figure 6c. CF/PPy‐NR/DHs under N_2_ exhibits an irreversible redox potential at around −0.2 V with the maximum current density of −1.5 mA/cm^2^ indicating polymer reduction. We observed that the correlated peak current was suppressed and slightly shifted to more negative potentials after purging the system with CO_2_. The current density at the range of −1.5 mA/cm^2^ is not large with the adherence of 3 enzymes onto the electrode. It can be attributed to the pyrrole monomers may not be able to fully diffuse on CF during polymerization in presence of enzymes. One of the hurdles to improve the efficiency of the electrocatalytic reduction of CO_2_ is the relatively low solubility of carbon dioxide in water at standard conditions.^[47]^ Introducing gaseous CO_2_ into the electrolyte at the active site through a gas diffusion is a critical factor since the process of CO_2_ adsorption is reversible. To overcome this challenge, the system is purged with CO_2_ gas for saturation.

Chronoamperometric electrolysis were performed for 16 h under the applied constant potential −1.20 V for CO_2_ conversion. Current‐time curves of chronoamperometric electrolysis is depicted in the supplementary information (Figure S4d.) Multi‐enzyme modified electrode was successfully utilized for CO_2_ conversion and the electrolysis confirms the methanol production. The CF/PPy‐NR/DHs electrode exhibited the FE up to 10 % for the methanol amount of ~7 ppm. The enzymes are active after immobilization presumably without any significant configurational changes during the reaction. The optimal pH values has been reported for FDH, F_al_DH, ADH involved in the sequential reaction to catalyze CO_2_ reduction.^[30]^ Our results indicate that the temperature and pH is within the optimal range (pH 6.0–8.1) to provide protective environment for sufficient enzymatic activity.

The FE is moderately lower than our previously published results with immobilized dehydrogenase enzymes on graphene‐based electrodes for CO_2_ reduction in 20 h.^[46]^ This can be explained with a different attachment mode, i. e. covalent bonding of dehydrogenases on the substrate. Comparing to ADH‐modified electrodes, the enzyme load is lower within the same matrix for the cascade system in which less enzymes molecules included in total concentration of enzyme solution. Furthermore, the proposed cascade reaction mechanism is predominantly dictated by the selectivity of the products generated at each step during the CO_2_ reduction. As previously reported, the reaction rate of the forward reaction of FDH is lower than the reverse reaction.^[48]^ After this step, F_al_DH is used as a catalyst to reduce formate to formaldehyde in the second step of cascade reaction. In particular, F_al_DH enzyme with low activity and high sensitivity to substrate/product concentration and pH, is the bottleneck of the 3‐enzyme system.^[49]^ As a result, both enzymes limit the reaction efficiency by retarding the forward reaction rate.[^30^, ^48^]

Since the reduction rate of CO_2_ to formate is the limiting initial step of the cascade reaction as mentioned above, the conversion of CO_2_ occurs slowly.^[48]^ To determine the operational efficiency and the catalytic performance over time, we further designed electrodes with FDH. The FDH‐modified electrodes were tested for CO_2_ reduction using the sacrificial cofactor NADH. The catalytic reaction was initiated by NADH (9 mg, ~0.13 mmol) addition and the liquid samples were collected after 13, 16, and 20 hours. The analysis of the products was carried out using ion chromatography (Figure S11) and the conversion efficiencies (CE) are shown in Table S2. The maximum CE of ~69 % was obtained for the chemical conversion of CO_2_ to formate using CF/PPy‐NR/FDH electrode for 20 h. Moreover, we performed electrolysis at applied constant potential of −1.2 V without adding NADH and the results are summarized in Figure S12 and Figure S13. The maximum FE of ~20 % was calculated for the electrochemical reduction of CO_2_ for the FDH‐modified electrode after 16 hours (Table S3). The data represents the catalytic activity of immobilized FDH and the product formation over time during chemical and electrochemical reduction of CO_2_.

Table 1 shows detailed information for enzyme based bioelectrodes fabrication using different immobilization methods. Bioelectrodes are towards CO_2_ reduction to formate and methanol production *via* direct (DET) or hybrid electron transfer mechanism (HET). The faradaic efficiency, potential, current density are summarized. The faradaic efficiency for formate (FE_formate_) could reach up to 100 % while it for methanol (FE_methanol_) was merely ~10–12 %, which can be attributed to insufficient mass transfer and electron transfer in cascade enzymatic reaction.^[50]^

**Table 1:** Enzyme modified bioelectrodes for CO_2_ conversion *via* DET and hybrid electron transfer mechanism.

Enzymes	Immobilization Method/Electrode	Product	%FE	Applied Potential	Current Density	Area	Stability	Reference
FDH	Adsorption/**Pyrolytic graphite edge electrode**	Formate	97.3 %	0.81 V vs. SHE	8.5 μA cm^−2^	3.94 cm^2^	–	Reda *et al*., 2008, [51]
FDH	Adsorption/**Graphite‐epoxy pot electrode**	Formate	101.7 %	−0.6 V vs. SHE	–	5.3 cm^2^	1–2 h	Bassegoda *et al*., 2014, [52]
FDH	Conjugation to conductive hydrogel/**Carbon clothe electrode**	Formate	92.7 %	−0.6 V vs. Ag/ AgCl	–	1 cm^2^	12 h	Kuk *et al*., 2019, [53]
FDH	Covalent immobilization/ **Gold and graphite electrodes**	Formate	100 %	−0.6 V vs. SHE	−160 μA cm^−2^	0.07 cm^2^	2 h	Alvarez *et al*., 2021, [54]
FDH	Immobilization on **inverse opal (IO**)**‐TiO_2_**	Formate	0.8 %	+0.4 V vs. SHE	−5 mA cm^−2^	0.19 cm^2^	10 h	Moore *et al*., 2021, [55]
FDH	Adsorption on **dye‐sensitized IO‐TiO_2_**	Formate	–	−0.6 V vs. SHE	−240 μA cm^−2^	0.25 cm^2^	2 h	Sokol *et al*., 2018, [56]
FDH, F_ald_DH, ADH	Entrapment in alginate–silicate hybrid gel matrix/**Carbon felt**	MeOH	10 %	−1.2 V vs. Ag/ AgCl	–	2x0.6x0.6 cm^3^	4 h	Schlager *et al*., 2016, [8]
FDH, F_ald_DH, ADH	Covalently bonding on graphene**/Carbon felt**	MeOH	12 %	−1.2 V vs. Ag/ AgCl	1 mA cm^−2^	2x0.6x0.6 cm^3^	20 h	Seelajaroen *et al*., 2020, [46]
FDH, F_ald_DH, ADH	Entrapment within conductive redox matrix/**Carbon felt**	MeOH	10 %	−1.2 V vs. Ag/ AgCl	1.5–3 mA cm^−2^	1 cm^2^	**16 h**	*This work (HET)

## 3 Conclusions

Novel enzyme‐based carbon felt electrodes were constructed and evaluated for electrocatalysis. We projected that the simultaneous electrodeposition of polypyrrole and neutral red in mild conditions will provide a simple approach for enzyme immobilization. The dehydrogenase enzymes and redox mediator were simultaneously entrapped into the polymer matrix. The modified electrodes were tested for single and multi‐enzymatic catalysis for ethanol and methanol production. CO_2_ reduction was conducted via 3‐enzyme cascade reaction (FDH, F_al_DH, ADH). Faradaic efficiencies 33 % and 10 % were calculated for the maximum ethanol and methanol production, respectively. In both cases, the proposed method permitted the catalytic activity of enzyme without using the cofactor NADH. The polymeric enzyme encapsulation method was successfully achieved to enable efficient electrochemical communication between redox enzymes and electrode surfaces.

The proposed method provides an efficient and simple in‐situ entrapment of enzymes within biocompatible conductive polymer matrix for rapid electron supply during hybrid electron transfer mechanism. In this hybrid mechanism, the conductive polymer acts as an electron‐conducting scaffold, and the immobilized mediator enhances the electron transfer. A facile one‐step synthesis in aqueous environment at room temperature enables sustainable process for cathode bio‐functionalization. We report stability of up to 16 hours for modified electrodes prepared through a one‐pot electrochemical approach. For future investigations in the field, the present work is considerably unique owing to porous CF electrode with the conductive polymer matrix comprising immobilized redox mediator. The procedure allows the substitution of natural cofactor NADH. Future work may focus on prolonged operational stability and reuse potential of the modified electrodes to explore effective lifetime for long‐term applications in biofuels and biosensors.

## Experimental Section

### Materials

All dehydrogenase enzymes: ADH (alcohol dehydrogenase from *Saccharomyces cerevisiae*); FDH (formate dehydrogenase from *Candida boidinii*), and F_al_DH (formaldehyde dehydrogenase from *Pseudomonas sp*.) were purchased from Sigma Aldrich. NaCl (ACM), KCl (Alfa Aesar), Na_2_HPO (Sigma Aldrich), KH_2_PO_4_ (Sigma Aldrich), were obtained from the relevant suppliers. All the other reagents, acetaldehyde, solvents were purchased from Sigma Aldrich.

Pyrrole and neutral red (3‐Amino‐7‐dimethylamino‐2‐methylphenazine hydrochloride) were procured from Sigma Aldrich. Pyrrole was distilled prior to use. All other chemicals were at analytical grade and used as such without further purification. The electropolymerization and electrolysis were performed using 18 MOhm ultrapure water (18 MΩ H_2_O).

Carbon felt electrodes were purchased from SGL Carbon GmbH. The phosphate buffer saline (PBS) electrolyte was prepared from appropriate mixture of NaCl (0.137 M), KCl (2.7 mM), Na_2_HPO_4_ (0.01 M), KH_2_PO_4_ (1.8 mM) with a pH value of 7.4.

### Synthesis of Polymer and Enzyme Entrapment

Carbon felt electrode with diameter of 0.8×0.5×0.2 cm^3^ was connected with a platinum (Pt) wire and rinsed with 18 MΩ water prior to use. The enzyme solutions were prepared separately for ethanol production and three enzymes cascade reaction.

6 mg ADH and 6 mg FDH were dissolved separately in 1 mL of 0.1 M PBS (pH 7.4) solutions and CF electrodes were merged into these enzyme solutions for 20 hours. The solution with 3‐enzymes was prepared by dissolving 5.8 mg of FDH, 1.4 mg of F_al_DH, and 1.4 mg of ADH in 1.2 mL of 0.1 M PBS (pH 7.4) solution to soak the electrode inside for 20 hours.

After, the carbon felt containing enzymes was inserted into the electrochemical cell with PBS electrolyte solution. Supporting electrolyte covered 1×1 cm^2^ of carbon felt electrode to secure the connecting Pt wire. A one compartment three–electrode cell was utilized for electropolymerization, consisting of carbon felt as working electrode, Ag/AgCl (3 M KCl) as reference, and Pt plate as counter electrode. Immobilization of enzyme was achieved via electropolymerization of conductive polymer on carbon felt substrate in 10 mL PBS solution (pH 7.4). The electropolymerized PPy was optimized by added monomer resulting in concentration of 0.3 M and 0.15 M of pyrrole. The emulsion was stirred vigorously prior to synthesis. In‐situ oxidative electropolymerization of 0.3 M PPy was conducted by cycling voltammetry with the sweeping voltage between −0.4 V and +1.0 V at a scan rate of 50 mV/s after 20 cycles at room temperature. PBS including 0.15 M pyrrole with addition of 0.2 mM or 0.4 mM neutral red (NR) was used for co‐deposition of pyrrole and NR. The simultaneous polymerization was carried out by cycling voltammetry at the potential range potential between −1.2 V and +1.0 V with a scan rate of 50 mV/s with cycle numbers 18 and 20 at room temperature.

Subsequently, enzyme‐modified CF electrodes were washed in deionized 18 MΩ H_2_O and 0.1 M PBS solution in consecutive steps. First, the electrode was washed after soaking CF electrode in 18 MΩ H_2_0 for approximately 15 minutes to remove residual compounds. This step was repeated three times by rinsing the electrode with 18 MΩ water after each step. Finally, CF electrode was kept in PBS solution for about 20–30 mins and rinsed with same electrolyte solution prior to electrolysis. The average washing time was ~1 h.

### Chemical Reactions

The ADH modified electrodes were transferred into a vial containing 4 mL of 0.1 M PBS buffer solution at pH 7.4. Subsequently, 1 mL of 0.3 M acetaldehyde solution was added to the electrolyte solution. The vial was then purged with N_2_ for 1 h and then 1 mL of solution was taken (T=0 sample – before adding) and 1 μmole of NADH (1 mL). The reaction was stirring gently. 1 mL samples were taken after 16 hours.

FDH modified electrodes were transferred into a vial containing 10 mL of 0.1 M PBS buffer solution at pH 7.4. The vial was then purged with CO_2_ for 1 h and then 0.2 mL of solution was taken (T=0 sample – before adding) and 0.2 mL of NADH solution was added (40.5 mg NADH dissolved in 0.9 mL PBS at pH 7.4). The reaction was stirring gently. 0.2 mL samples were taken after 13, 16, and 20 hours.

### Electrochemical Studies

All the electrolysis procedures were completed in a two‐compartment electrochemical cell (H‐cell) equipped with three‐electrode system. The electrolysis was performed in H‐cell including 20 mL 0.1 M PBS (pH 7.4) electrolyte solution in anode and cathode sides which were separated with a glass frit. Enzyme immobilized CF working electrode and an Ag/AgCl reference electrode were inserted into cathode side, while a Pt electrode was employed as counter electrode in anode side.

Prior to electrolysis, cyclic voltammograms were recorded at applied potentials between 0 and −1.20 V with a scan rate of 25 mV/s in the presence of 1 M acetaldehyde under N_2_‐saturated conditions for the reduction of acetaldehyde. In case of investigation of CO_2_ conversion, the voltammograms were recorded under CO_2_‐saturated condition after purging the system with CO_2_ for 2 hours.

Chronoamperometric electrolysis were performed in 0.1 M PBS for 16 h under the applied constant potentials −1.0 V for the reduction of acetaldehyde and −1.20 V for CO_2_ conversion by using the electrochemical setup at room temperature with gentle stirring. Before adding the reactants (control), in the beginning of the reduction (T=0), and after reduction completed (T=16 h) 1 mL samples were collected for analysis. The samples were analyzed by using liquid‐injection gas chromatography and products are detected in ppm levels.

The Faradaic Efficiency (FE) is calculated using following formula:

$$\mathrm{FE}\;\left(\%\right)=\frac{\mathrm{moles}\;\mathrm{of}\;\mathrm{product}\left(s\right)}{\;\frac{1}{n}•moles\;of\;electron}\;•100$$

where *n* is the number of electrons required for the reduction process. 2 electron is involved in the reduction of acetaldehyde to ethanol, while conversion of CO_2_ to methanol demands 6 electrons in total for the cascade reaction. Moles of products are calculated from the amount of ethanol/methanol produced during electrolysis. Moles of electrons are quantified by dividing the number of injected charges during electrolysis by the Faradaic constant of 96485.33 C/mol.

### Analytical Methods

The product analysis for ethanol and methanol was performed via GC by using a Thermo Scientific Trace 1300 **Gas Chromatograph** employing a TR−V1 column and equipped with a flame ionization detector (FID). The samples were filtered through regenerated cellulose (RC) membrane filters with 0.45 μm pore size prior injecting to remove potential particles. The formate product analysis was carried out *via* **Ion chromatography (IC**) technique by using Thermo Scientific Dionex ICS‐5000 ion chromatograph (IC) equipped with IonPac AG19 guard column (2×50 mm), an IonPac AS19 column (2×250 mm) and a Dionex suppressor‐conductivity detector. The formate samples were diluted with ultrapure deionized water (with the ratio of 1 : 50) and the measurements were carried out using an autosampler at a constant temperature of 25 °C.

**Scanning electron microscope** (**SEM**) measurements were performed with JEOL JSM‐6360 LV scanning electron microscope with an electron beam accelerating voltage of 7 kV with a working distance of 12 mm. For the SEM investigation, the samples were prepared by cutting the upper part of each electrode where attached to Pt wire. Bottom part of the sample (with size of ~1x1 cm^2^) was dried under ambient conditions overnight.

**X‐ray photoelectron spectroscopy** (**XPS**) measurements were performed on a Theta Probe XPS‐system (Thermofisher, GBR), which features a monochromated Al−Kα X‐ray source with an energy of 1486.6 eV. The spot size on the sample surface was 400 μm in diameter. The hemispherical analyzer was set to a pass energy of 20 eV for the recorded high–resolution (HR) scans at an energy step size of 0.05 eV. The data acquisition and evaluation, as well as the control of the device, are performed *via* a software package (Avantage) provided by the system manufacturer.

**Raman spectroscopy** was conducted using a Bruker MultiRAM FT‐Raman spectrometer with an excitation wavelength of 1064 nm in combination with a liquid nitrogen‐cooled Ge detector. The spectra were recorded with a resolution of 4 cm^−1^ and by averaging of 1000 scans.

The samples for XPS and Raman spectroscopy were prepared analogous to the electropolymerizations at CF on indium tin oxide (ITO) coated glass and platinum electrode sheets, respectively. Prior to deposition, the ITO‐coated glass was cleaned with acetone and isopropanol in an ultrasonic bath for 15 min each and consequently blow‐dried with N_2_ gas. The monomer ratio, sweeping potentials, sweeping rate and cycle numbers were kept identical to deposit the respective material. Raman spectra of neutral red was recorded by using its powder.

**UV‐Vis spectroscopy**: The absorption spectra were measured at room temperature on a PerkinElmer Lambda 1050 UV/Vis/NIR spectrophotometer. The washing solution of modified electrode (CF/PPy‐NR) and the sample solution after completed electrolysis (PBS aqueous solution) were measured to control for potentially leached neutral red.

## Author Contributions

CRediT authorship contribution statement: H.S. investigation, methodology; F.M. investigation (Raman, XPS), writing – review & editing; D.W. investigation, writing – review & editing; M.C. investigation (XPS); C.U. investigation, writing – review & editing; N.S.S. conceptualization, project administration, funding acquisition, writing – review & editing; S.T. conceptualization, project administration, funding acquisition, methodology, investigation, visualization, writing – original draft, writing – review & editing.

## Conflict of Interests

The authors declare no conflict of interest.

4

## Supporting information

As a service to our authors and readers, this journal provides supporting information supplied by the authors. Such materials are peer reviewed and may be re‐organized for online delivery, but are not copy‐edited or typeset. Technical support issues arising from supporting information (other than missing files) should be addressed to the authors.

Supporting Information

## Data Availability

The data that support the findings of this study are available from the corresponding author upon reasonable request.
