# Supplementary material for: Sustainable One‐Pot Electrochemical Approach for Entrapment of Multi‐Enzymes and Biocompatible Redox Mediator
Source: Chempluschem. 2025 Feb 20;90(5):e202400577. doi: 10.1002/cplu.202400577 (PMC12105433; doi:10.1002/cplu.202400577)
Supplement: Supplementary file 1 — Supporting Information [file CPLU-90-e202400577-s001.pdf]

ChemPlusChem

Supporting Information

# Sustainable One-Pot Electrochemical Approach for Entrapment of Multi-Enzymes and Biocompatible Redox Mediator

Hathaichanok Seelajaroen, Felix Mayr, Dominik Wielend, Munise Cobet, Christoph Ulbricht, Niyazi Serdar Sariciftci, and Serpil Tekoglu*

# Supplementary Information

## Sustainable One-Pot Electrochemical Approach for Entrapment of Multi-Enzymes and Biocompatible Redox Mediator

*Hathaichanok Seelajaroen, Felix Mayr, Dominik Wielend, Munise Cobet, Christoph Ulbricht, Niyazi Serdar Sariciftci, Serpil Tekoglu**

Linz Institute for Organic Solar Cells (LIOS), Institute of Physical Chemistry, Johannes Kepler University Linz, Altenberger Str 69, A-4040, Linz, Austria.

**Corresponding author: serpil.tekoglu@jku.at*

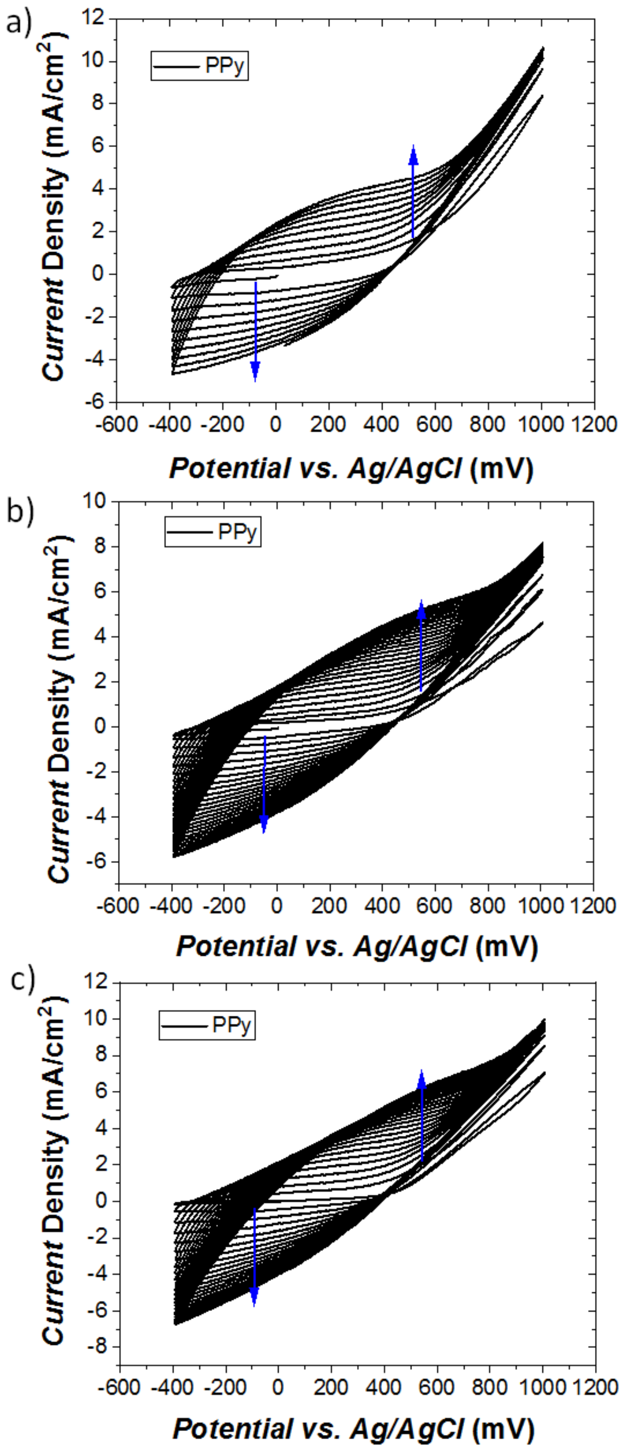


**Fig. S1.** Cyclic voltammograms of PPy deposition on carbon felt (CF) electrode in 0.1 M PBS (pH 7.4) with **a)** 0.15 M pyrrole, 10 cycles, **b)** 0.15 M pyrrole, 25 cycles, and **c)** 0.3 M pyrrole, 25 cycles, each at a sweep rate of 50 mV/s.

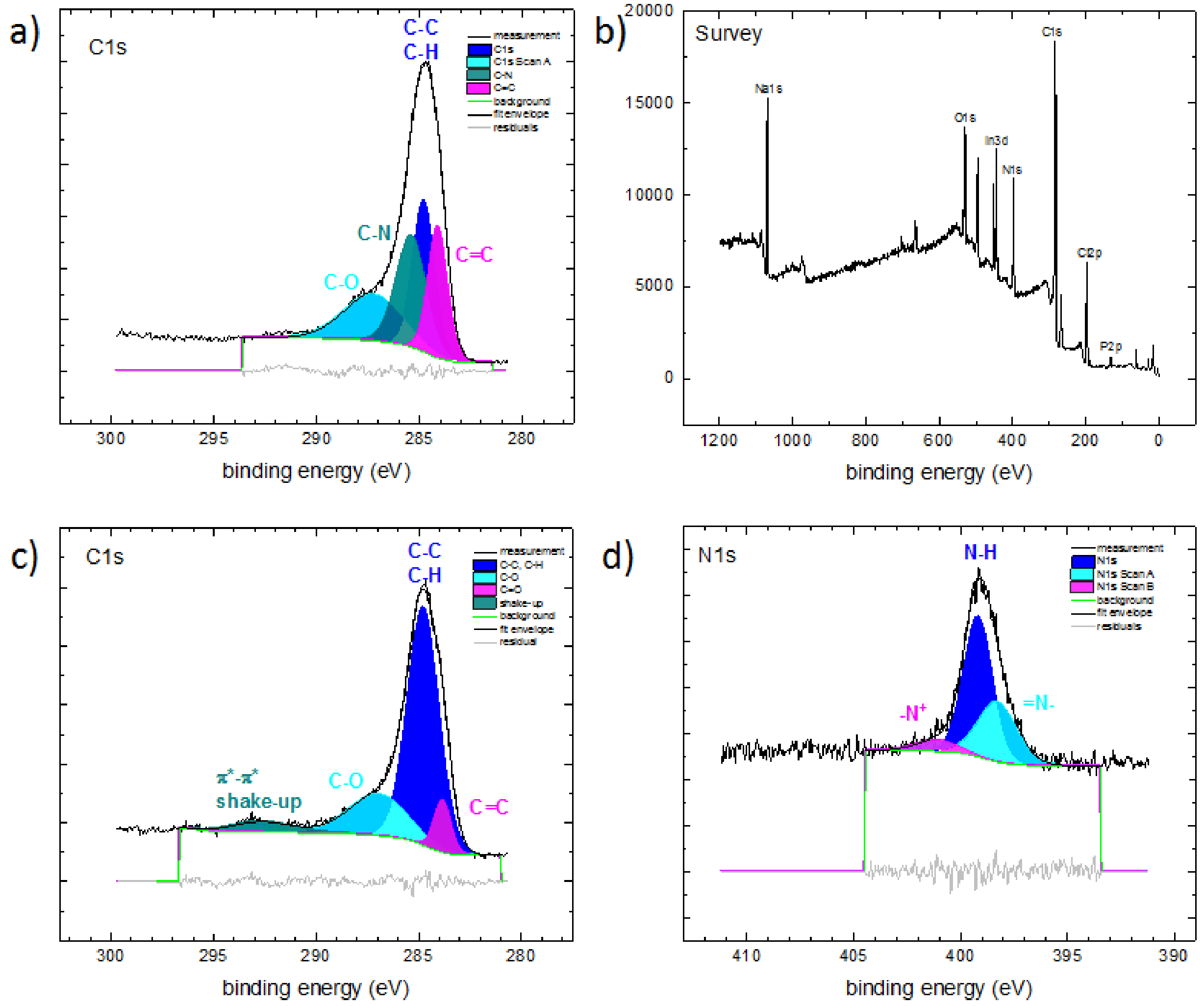


**Fig. S2.** XPS spectrum of PPy and PPy-NR. **a)** C1s deconvolution of pristine PPy, **b)** Survey and **c)** C1s deconvolution of PPy-NR (0.4 mM). **d)** N1s deconvolution of of PPy-NR (0.2 mM).

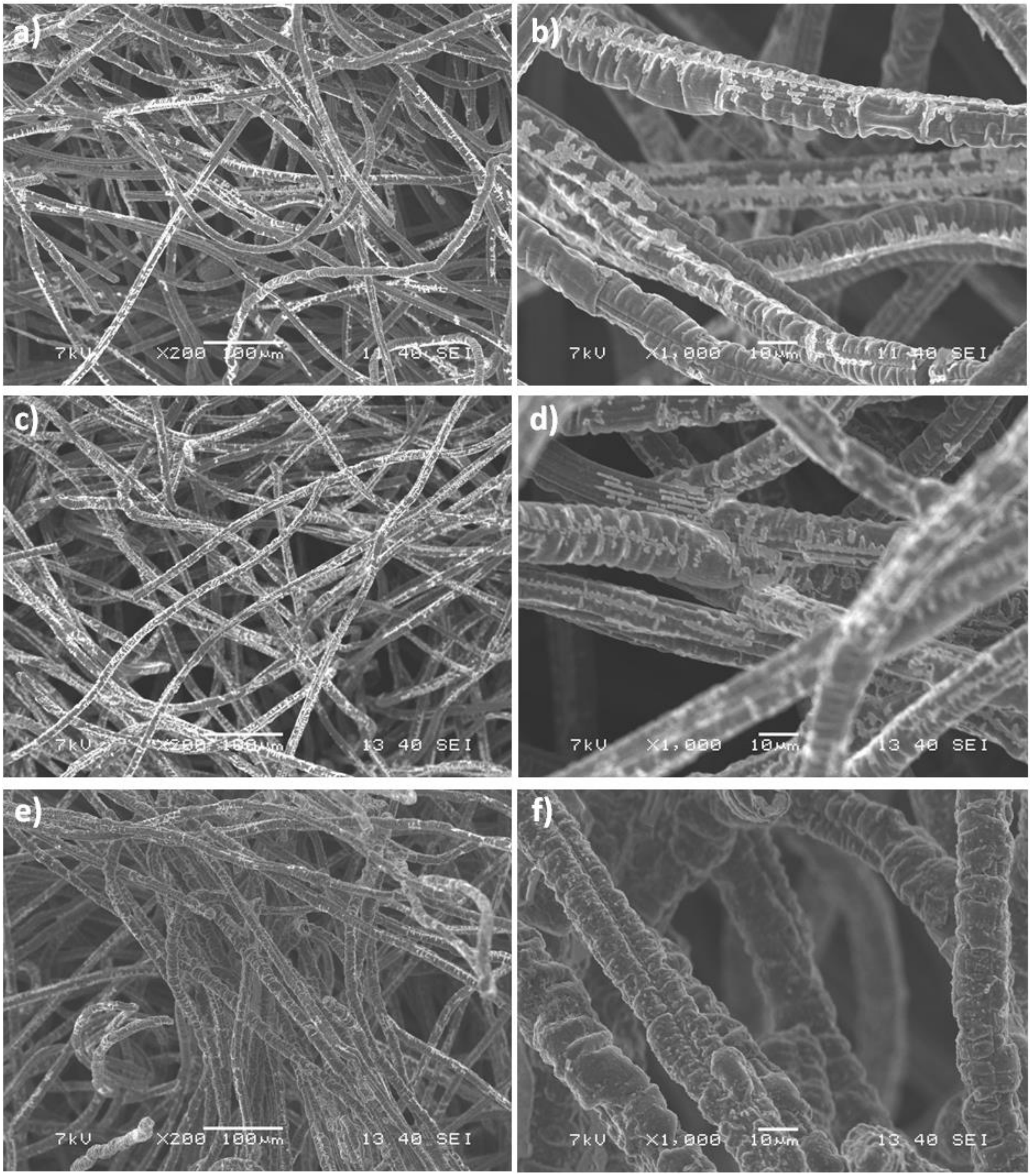


**Fig. S3.** SEM images of CF/PPy/ADH with 200× and 1,000× magnifications after different washing time: **a-b)** washed one hour in PBS, **c-d)** washed 24 hours in PBS. **e-f)** CF/PPy/ADH electrode after 16 h electrolysis in PBS.

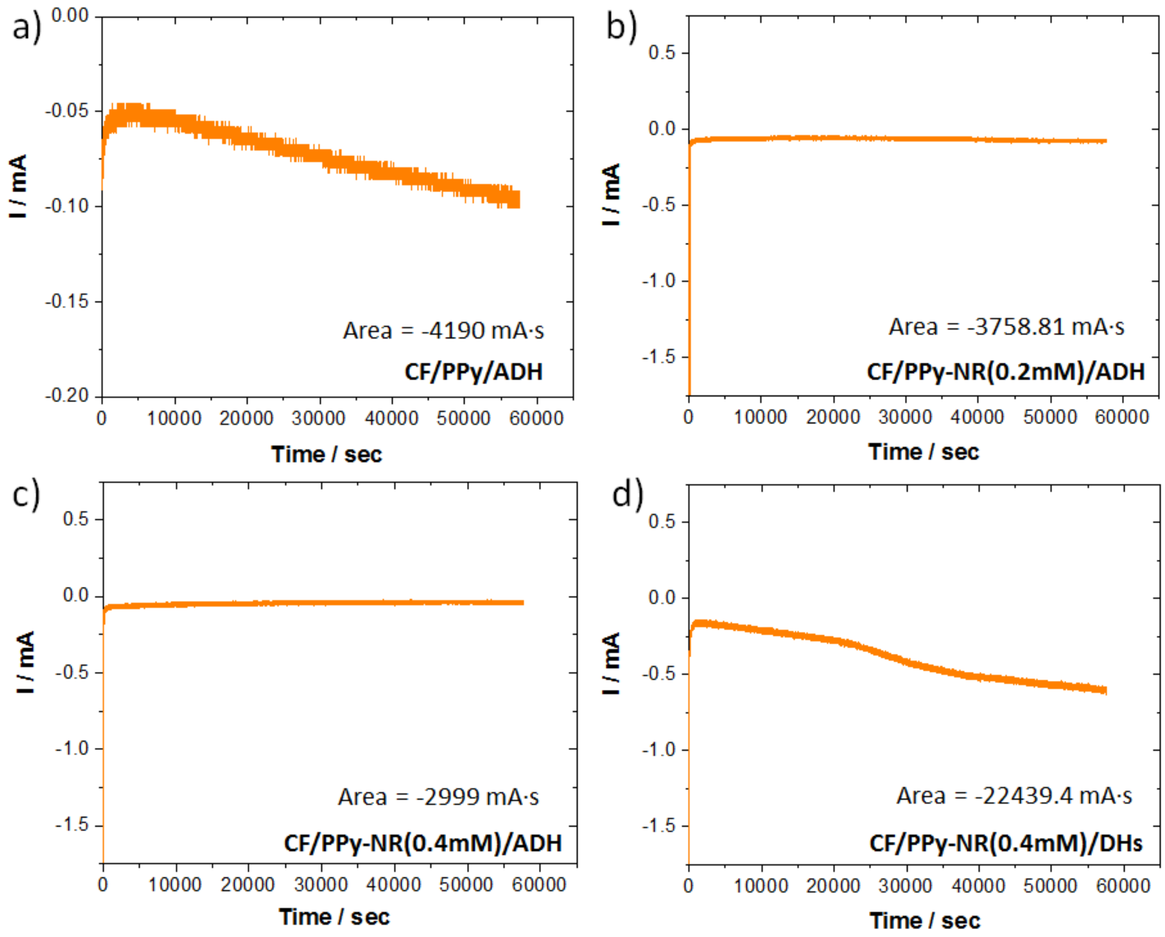


**Fig. S4. a)** Current-time curves of chronoamperometric electrolysis at the applied constant potential of **a-c)** −1.0 V for acetaldehyde reduction and **d)** −1.2 V for $CO_2$ reduction for 16 h. Integrated area is inserted on the graph for each respective modified electrode.

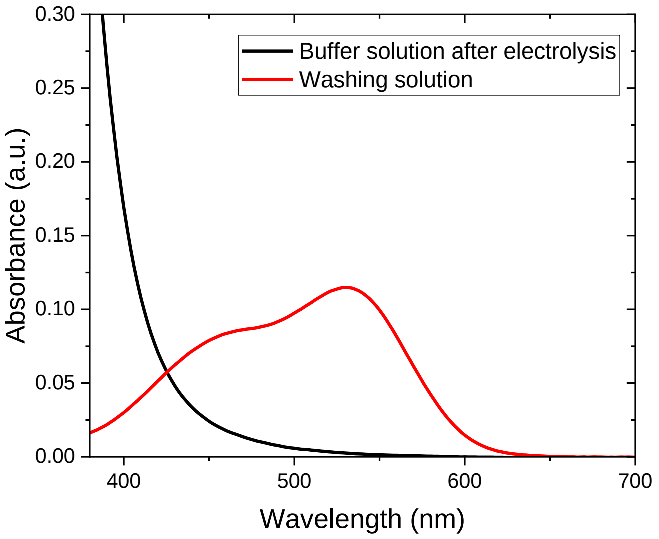


**Fig. S5.** UV-Vis spectra for the washing solution (after one hour) of modified electrode CF/PPy-NR/ADH and the sample solution after completed electrolysis.

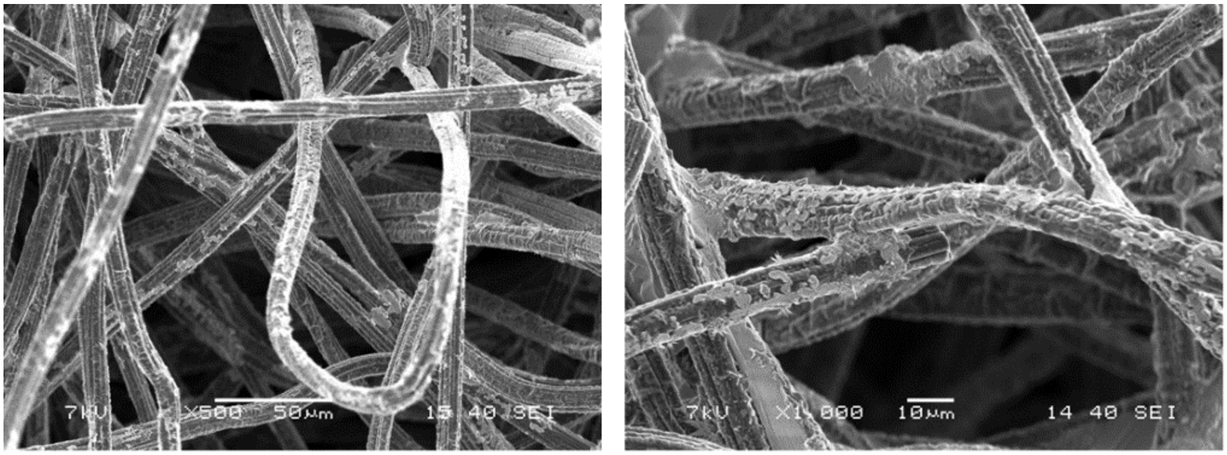


**Fig. S6.** SEM images of CF/PPy-NR/DHs with 500× and 1,000× magnifications. Electrodeposition of 0.15 M pyrrole and 0.4 mM neutral red in 0.1 M PBS after 18 CV cycles.

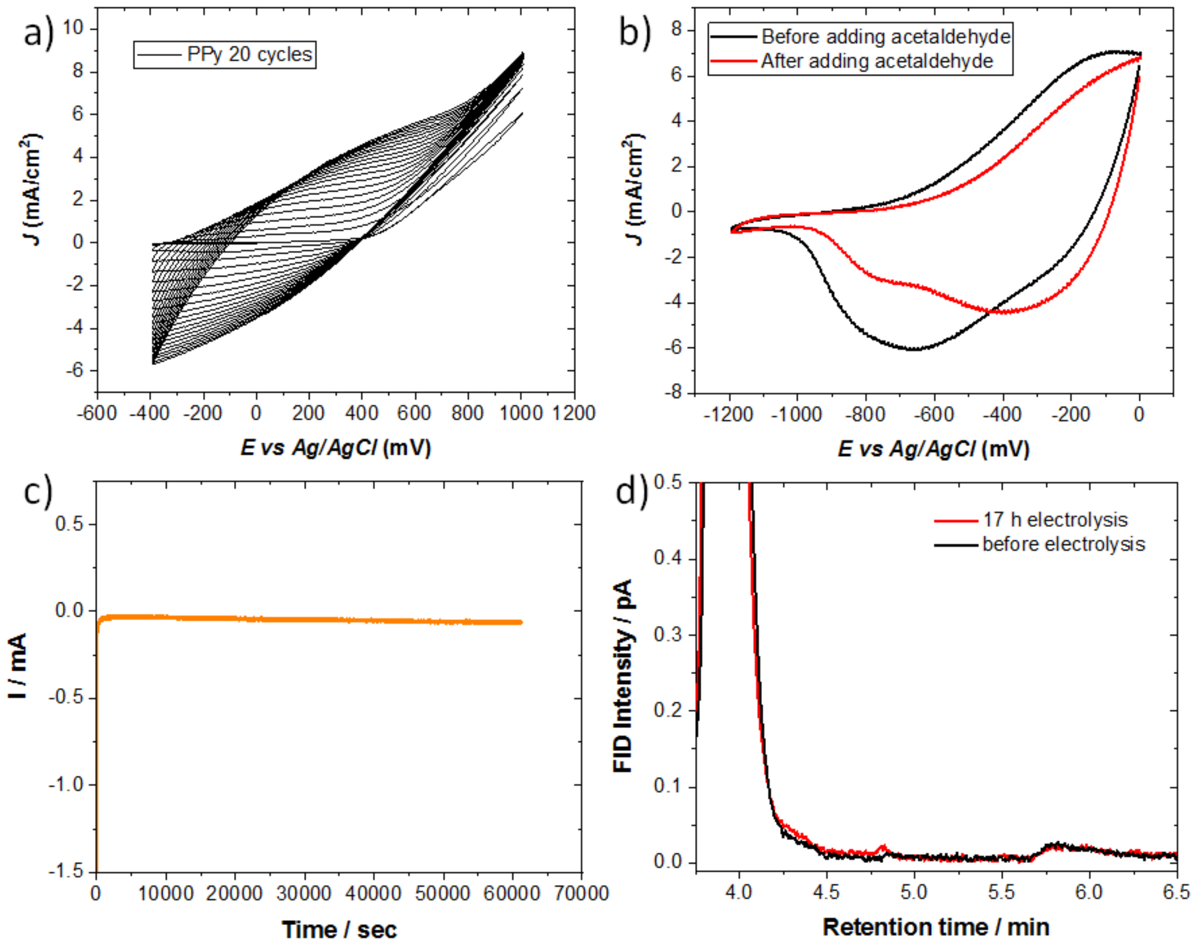


**Fig. S7.** Preparation and characterization of CF/PPy control electrodes without enzyme. **a)** Electropolymeriyation of 0.3 M pyrrole on CF in 0.1 M PBS (pH 7.4) by sweeping the voltage between -0.4 V and 1.0 V with the scan rate of 50 mV/s after 20 cycles. **b)** Electrochemical analysis of CF/PPy electrode at applied potential between 0 V and 1.2 V before and after adding the substrate (in $N_2$ saturation) **c)** Current-time curve of chronoamperometric electrolysis at the applied constant potential of −1.0 V for 17 h. **d)** Chromatograms from liquid GC for the methanol production analysis before and after electrolysis.

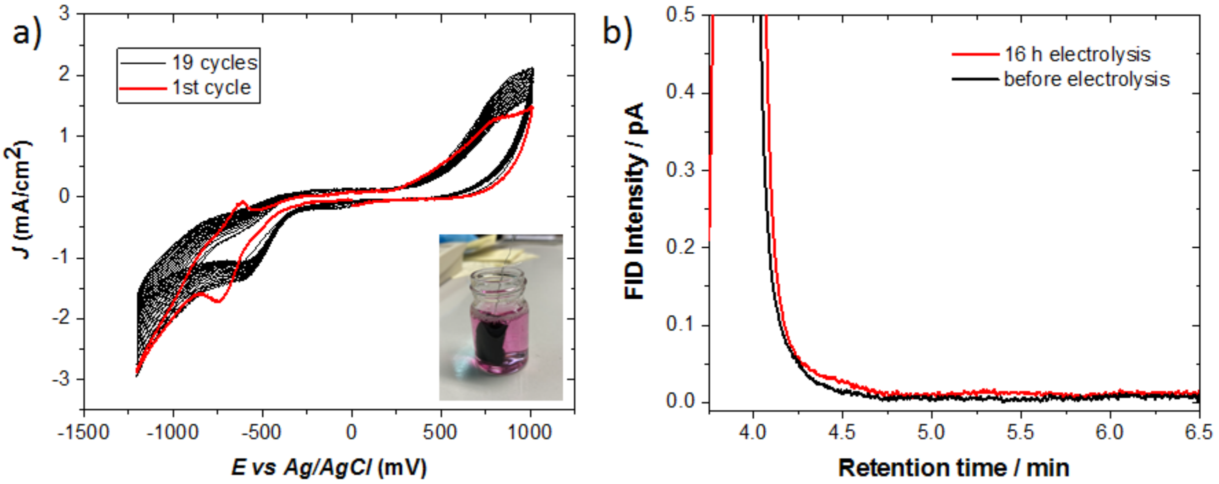


**Fig. S8.** Preparation and investigation of CF/NR/ADH. a) Electropolymerization of 1 mM neutral red on CF in 0.1 M PBS (pH 7.4) by sweeping the voltage between -1.2 V and 1.0 V with the scan rate of 50 mV/s after 20 cycles. b) Chromatograms from liquid GC for the ethanol production analysis after 16 h electrolysis. Inset image; washing step of the modified electrode in PBS.

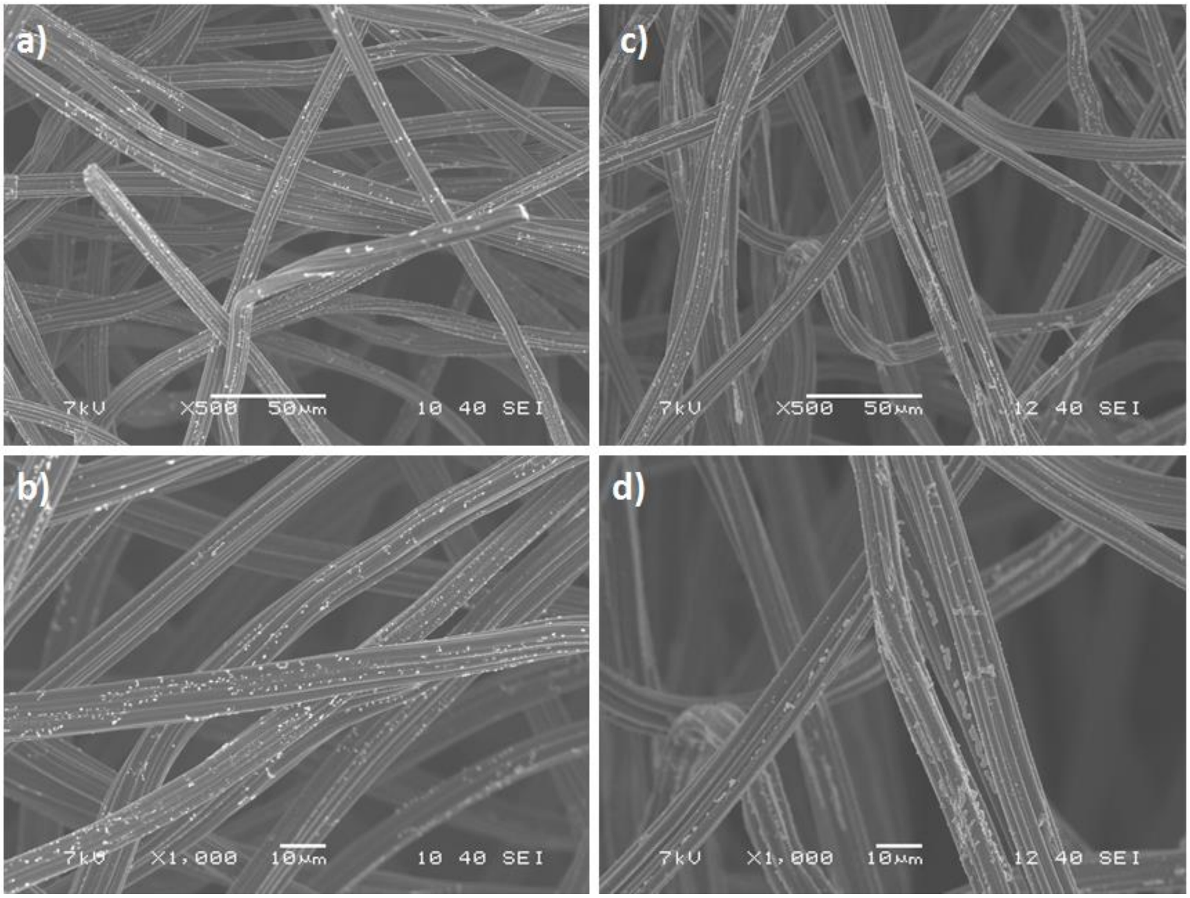


**Fig. S9.** SEM images of a-b) CF/NR and c-d) CF/NR/ADH with 500× and 1,000× magnifications.

## Chemical Reduction of Acetaldehyde to Ethanol:

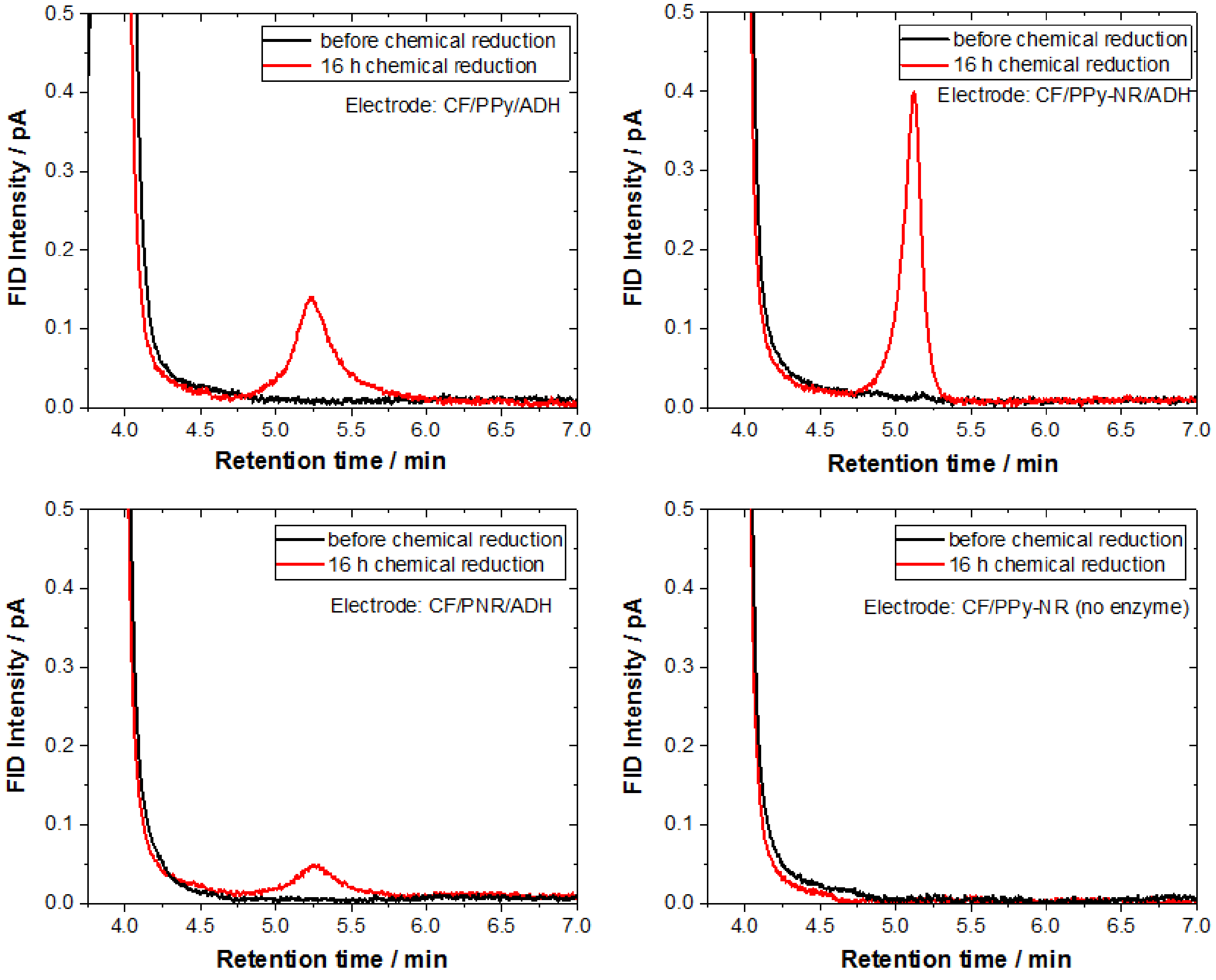


**Fig. S10.** Chromatograms from liquid GC for the ethanol production analysis after 16 h chemical reduction of acetaldehyde in the presence of NADH by using the modified electrodes: a) CF/PPy/ADH, b) CF/PPy-NR/ADH, c) CF/poly(neutral red)/ADH, and d) CF/PPy-NR without enzyme.

The chemical conversion efficiency (CE) is calculated according to the following equation below and the results are summarized in **Table S1**.

$$\mathrm{CE}\ (\%) = \frac{moles\ of\ product(s)}{moles\ of\ NADH} \cdot 100$$

where *moles of product* is calculated from the amount of ethanol produced during the chemical reduction and *moles of NADH* is calculated from the amount of NADH added into the system. To reduce one molecule of acetaldehyde to one molecule of ethanol, one molecule of NADH is involved in the reaction that converts NADH into $NAD^+$.

A 30% conversion efficiency was calculated for the chemical conversion of acetaldehyde to ethanol using CF electrodes with immobilized ADH in polypyrrole matrix. The CE was increased to 41% with a simultaneous co-immobilization of neutral red (NR) on the modified electrodes with ADH. To evaluate any possible catalytic contribution from pristine NR to immobilize ADH units on carbon felt, a control sample was prepared by electropolymerization of 1 mM NR to encapsulate enzyme on CF. A CE of 5.8% was obtained for the control sample of CF/PNR/ADH, showing that the pristine poly(neutral red) was not sufficient to keep the enzyme fixed on the electrode surface. The obtained results further verified that the catalytic activity of CF/PPy-NR/ADH was indeed the outcome of the hybrid electron transfer mechanism through immobilization of the enzyme on CF. Another control sample CF/PPy-NR without added ADH exhibited no product formation.

**Table S1.** Chemical conversion efficiencies toward reduction of acetaldehyde to ethanol using NADH as sacrificial cofactor for 16 hours.

| Electrode | Ethanol / ppm | Conversion Efficiency / % |
|---|---|---|
| CF/**PPy**/ADH | 14 | 30 |
| CF/**PPy-NR**/ADH | 19 | 41.2 |
| CF/**PNR**/ADH (control) | 2.7 | 5.8 |
| CF/**PPy-NR**/no enyzeme (control) | - | - |

## Chemical Reduction of $CO_2$ to Formate:

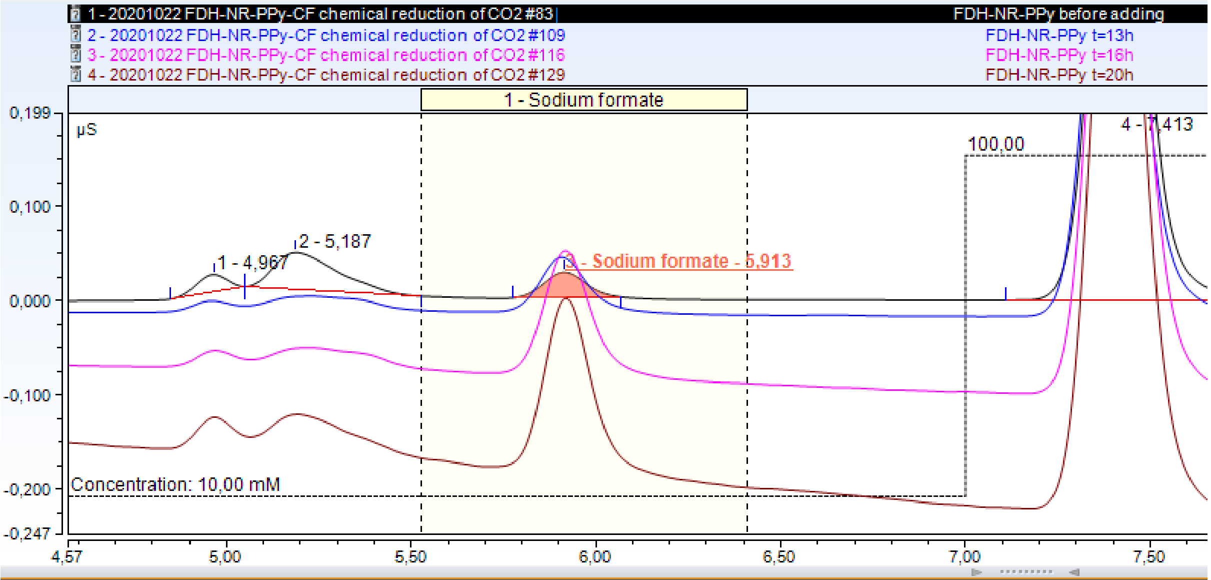


| | A | B | C | D | E | F | G | H |
|---|---|---|---|---|---|---|---|---|
| 1 | Inj. | Injection Name | Type | Ret.Time | Amount | Amnt.Dev. | Rel.Area | Area |
| 2 | No. | Selected Peak: | | min | µM | µM | % | µS*min |
| 3 | | Sodium formate | | Sodium forma | Sodium formate | Sodium forma | Sodium formate | Sodium forma |
| 4 | | | | CD_1 | CD_1 | CD_1 | CD_1 | CD_1 |
| 5 | 83 | FDH-NR-PPy before adding | Unknown | 5,913 | 11,6806 | n.a. | 0,01 | 0,0034 |
| 6 | 109 | FDH-NR-PPy t=13h | Unknown | 5,907 | 30,0086 | n.a. | 0,05 | 0,0092 |
| 7 | 116 | FDH-NR-PPy t=16h | Unknown | 5,917 | 66,7879 | n.a. | 0,08 | 0,0207 |
| 8 | 129 | FDH-NR-PPy t=20h | Unknown | 5,920 | 93,7564 | n.a. | 0,11 | 0,0291 |

**Fig. S11.** Chromatograms from IC for the formate production analysis after 20 h chemical reduction of $CO_2$ in the presence of NADH by using the modified electrode CF/PPy-NR/FDH. Column E shows the corresponding formate amounts (in micromoles) for each sample.

The CE is calculated using same chemical conversion equation above where *moles of product* is calculated from the amount of *formate* produced during the chemical reduction and *moles of NADH* added into the system. The results are shown in **Table S2**. One molecule of NADH is utilized to reduce one molecule of $CO_2$ to one molecule of formate.

The conversion efficiency of ~49% was calculated for the chemical conversion of $CO_2$ to formate using CF electrodes with immobilized FDH after 16 hours. We observed that the amount of the product increased by the reaction time and reached the maximum at 20 h. The CE was enhanced to ~69% with a simultaneous co-immobilization of neutral red (NR) on the modified electrode. There is a clear upward trend over time, with the conversion rate steadily increasing from T=0 to T=20 h for the modified electrode. The data represents the catalytic activity of immobilized FDH and the product formation over time during chemical reduction of $CO_2$.

**Table S2.** Chemical conversion efficiencies for reduction of $CO_2$ to formate using FDH enyzme and NADH as sacrificial cofactor for 20 hours.

| Electrode | Reaction Time / hour | Formate / µmole | Conversion Efficiency / % |
|---|---|---|---|
| CF/**PPy-NR**/FDH | T=0 | 11.68 | 8.6 |
| | T=13 h | 30.00 | 22.2 |
| | T=16 h | 66.78 | 49.4 |
| | T=20 h | 93.75 | 69.4 |

NADH amount= 9 mg (~ 0.135 mmol).

## Electrochemical Reduction of $CO_2$ to Formate:

We further conducted electrolysis using CF/PPy-NR/FDH without introducing NADH into the system. The faradaic efficiency (FE) is calculated using the equation where *moles of product* is calculated from the amount of produced *formate* and *moles of electrons* injected during the electrolysis. 2 electrons are involved to reduce one molecule of $CO_2$ to one molecule of formate. The results are shown in **Table S3**. The maximum Faradaic Efficiency of ~20% was calculated for the electrochemical conversion of $CO_2$ to formate after 16 hours. We observed that the amount of the product increased by the reaction time. However, the FE dropped to 17.5% after 20 h electrolysis.

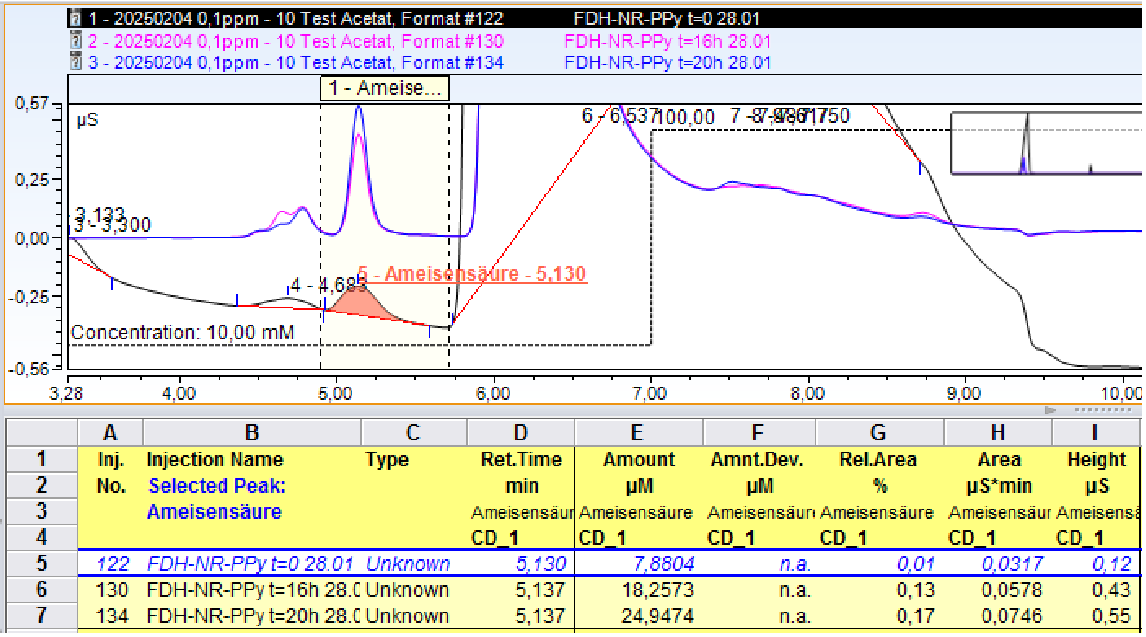


| | A | B | C | D | E | F | G | H | I |
|---|---|---|---|---|---|---|---|---|---|
| 1 | Inj. | Injection Name | Type | Ret.Time | Amount | Amnt.Dev. | Rel.Area | Area | Height |
| 2 | No. | Selected Peak: | | min | µM | µM | % | µS*min | µS |
| 3 | | Ameisensäure | | Ameisensäur | Ameisensäure | Ameisensäur | Ameisensäure | Ameisensäur | Ameisensä |
| 4 | | | | CD_1 | CD_1 | CD_1 | CD_1 | CD_1 | CD_1 |
| 5 | 122 | FDH-NR-PPy t=0 28.01 | Unknown | 5,130 | 7,8804 | n.a. | 0,01 | 0,0317 | 0,12 |
| 6 | 130 | FDH-NR-PPy t=16h 28.0 | Unknown | 5,137 | 18,2573 | n.a. | 0,13 | 0,0578 | 0,43 |
| 7 | 134 | FDH-NR-PPy t=20h 28.0 | Unknown | 5,137 | 24,9474 | n.a. | 0,17 | 0,0746 | 0,55 |

**Fig. S12.** Chromatograms from IC for the formate production analysis after 20 h electrochemical reduction of $CO_2$ by using the modified electrode CF/PPy-NR/FDH. Column E shows the corresponding formate amounts (in micromoles) for each sample. (Ameisensäure= formate)

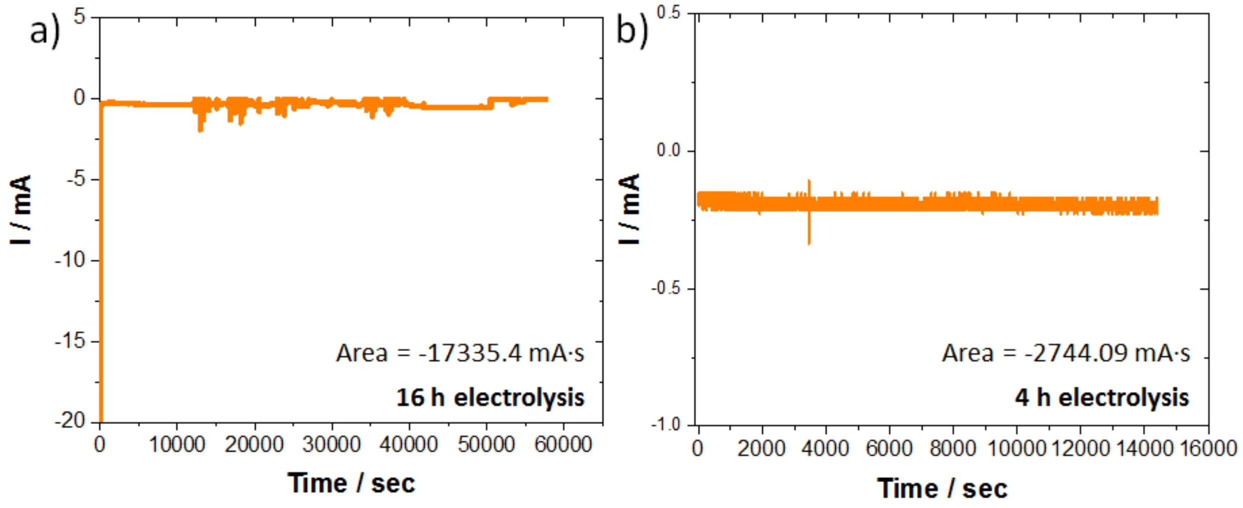


**Fig. S13.** Current-time curves of chronoamperometric electrolysis at the applied constant potential of −1.2 V for $CO_2$ reduction for **a)** the first 16 h and **b)** additional 4 h. Overall reaction time= 20 h. Integrated area is inserted on the graph for each respective modified electrode.

**Table S3.** Faradaic efficiencies for reduction of $CO_2$ to formate using FDH enyzme for 20 hours.

| Electrode | Reaction Time / hour | Formate / μmole | Faradaic Efficiency / % |
|---|---|---|---|
| CF/**PPy-NR**/FDH | T=0 | 7.88 | - |
| | T=16 h | 18.25 | 20.3 |
| | T=20 h | 24.94 | 17.5 |

In **Fig. S12**, the retention time of formate peak has been shifted to ~ 5.14-5.2 min after the reparation of IC. The reference calibration peak of pristine formate is shown in **Fig. S14** as indication of corresponding shift.

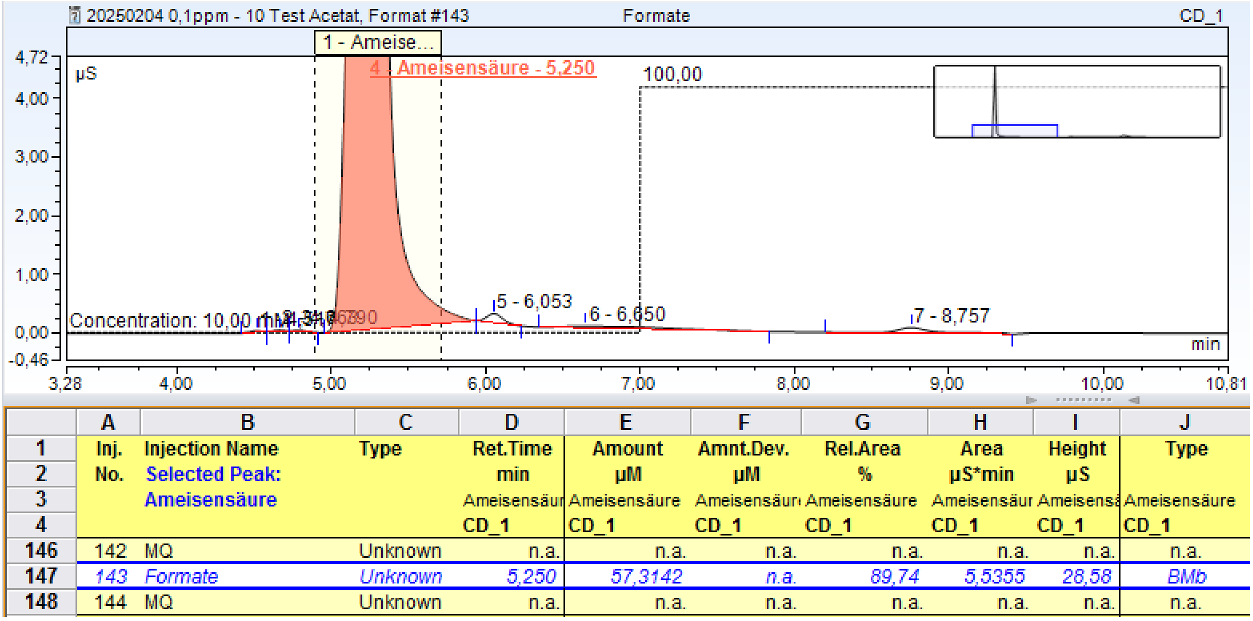


| | A | B | C | D | E | F | G | H | I | J |
|---|---|---|---|---|---|---|---|---|---|---|
| 1 | Inj. | Injection Name | Type | Ret.Time | Amount | Amnt.Dev. | Rel.Area | Area | Height | Type |
| 2 | No. | Selected Peak: | | min | µM | µM | % | µS*min | µS | |
| 3 | | Ameisensäure | | Ameisensäur | Ameisensäure | Ameisensäur | Ameisensäure | Ameisensäur | Ameisensä | Ameisensäure |
| 4 | | | | CD_1 | CD_1 | CD_1 | CD_1 | CD_1 | CD_1 | CD_1 |
| 146 | 142 | MQ | Unknown | n.a. | n.a. | n.a. | n.a. | n.a. | n.a. | n.a. |
| 147 | *143* | *Formate* | *Unknown* | *5,250* | *57,3142* | *n.a.* | *89,74* | *5,5355* | *28,58* | *BMb* |
| 148 | 144 | MQ | Unknown | n.a. | n.a. | n.a. | n.a. | n.a. | n.a. | n.a. |

**Fig. S14.** Chromatogram from IC for the pristine formate analysis as reference. (Ameisensäure= formate)
